# *NICOTIANAMINE SYNTHASE* activity affects nucleolar iron accumulation and impacts rDNA silencing and RNA methylation in Arabidopsis

**DOI:** 10.1093/jxb/erad180

**Published:** 2023-05-14

**Authors:** Charlotte Montacié, Christophe Riondet, Lili Wei, Tommy Darrière, Alizée Weiss, Frédéric Pontvianne, Marie-Line Escande, Anne de Bures, Edouard Jobet, Adrien Barbarossa, Marie-Christine Carpentier, Mark G M Aarts, Aurore Attina, Christophe Hirtz, Alexandre David, Virginie Marchand, Yuri Motorin, Catherine Curie, Stéphane Mari, Jean-Philippe Reichheld, Julio Sáez-Vásquez

**Affiliations:** Laboratoire Génome et Développement des Plantes (LGDP), UMR 5096, CNRS, 66860 Perpignan, France; LGDP, UMR 5096, Université Perpignan Via Domitia, 66860 Perpignan, France; Laboratoire Génome et Développement des Plantes (LGDP), UMR 5096, CNRS, 66860 Perpignan, France; LGDP, UMR 5096, Université Perpignan Via Domitia, 66860 Perpignan, France; Institut Agro, BPMP, CNRS, INRAE, Université Montpellier, 34060 Montpellier, France; Laboratoire Génome et Développement des Plantes (LGDP), UMR 5096, CNRS, 66860 Perpignan, France; LGDP, UMR 5096, Université Perpignan Via Domitia, 66860 Perpignan, France; Laboratoire Génome et Développement des Plantes (LGDP), UMR 5096, CNRS, 66860 Perpignan, France; LGDP, UMR 5096, Université Perpignan Via Domitia, 66860 Perpignan, France; Laboratoire Génome et Développement des Plantes (LGDP), UMR 5096, CNRS, 66860 Perpignan, France; LGDP, UMR 5096, Université Perpignan Via Domitia, 66860 Perpignan, France; Observatoire Océanologique de Banyuls s/ mer, CNRS, 66650 Banyuls-sur-mer, France; BioPIC Platform of the OOB, 66650 Banyuls-sur-mer, France; Laboratoire Génome et Développement des Plantes (LGDP), UMR 5096, CNRS, 66860 Perpignan, France; LGDP, UMR 5096, Université Perpignan Via Domitia, 66860 Perpignan, France; Laboratoire Génome et Développement des Plantes (LGDP), UMR 5096, CNRS, 66860 Perpignan, France; LGDP, UMR 5096, Université Perpignan Via Domitia, 66860 Perpignan, France; Laboratoire Génome et Développement des Plantes (LGDP), UMR 5096, CNRS, 66860 Perpignan, France; LGDP, UMR 5096, Université Perpignan Via Domitia, 66860 Perpignan, France; Laboratoire Génome et Développement des Plantes (LGDP), UMR 5096, CNRS, 66860 Perpignan, France; LGDP, UMR 5096, Université Perpignan Via Domitia, 66860 Perpignan, France; Laboratory of Genetics, Wageningen University & Research, 6700AA Wageningen, Netherlands; INSERM, CHU Montpellier, CNRS, IRMB, Université Montpellier, 34090 Montpellier, France; INSERM, CHU Montpellier, CNRS, IRMB, Université Montpellier, 34090 Montpellier, France; IGF, CNRS, INSERM, Université Montpellier, 34090 Montpellier, France; Epitranscriptomics and RNA Sequencing (EpiRNA-Seq) Core Facility, CNRS, INSERM, IBSLor (UMS2008/US40), Université de Lorraine, F-54000 Nancy, France; Epitranscriptomics and RNA Sequencing (EpiRNA-Seq) Core Facility, CNRS, INSERM, IBSLor (UMS2008/US40), Université de Lorraine, F-54000 Nancy, France; CNRS, IMoPA (UMR 7365), Université de Lorraine, F-54000 Nancy, France; Institut Agro, BPMP, CNRS, INRAE, Université Montpellier, 34060 Montpellier, France; Institut Agro, BPMP, CNRS, INRAE, Université Montpellier, 34060 Montpellier, France; Laboratoire Génome et Développement des Plantes (LGDP), UMR 5096, CNRS, 66860 Perpignan, France; LGDP, UMR 5096, Université Perpignan Via Domitia, 66860 Perpignan, France; Laboratoire Génome et Développement des Plantes (LGDP), UMR 5096, CNRS, 66860 Perpignan, France; LGDP, UMR 5096, Université Perpignan Via Domitia, 66860 Perpignan, France; Instituto de Agrobiotecnología del Litoral, Argentina

**Keywords:** Iron, methylation, nicotianamine, nucleolus, rDNA, redox

## Abstract

In plant cells, a large pool of iron (Fe) is contained in the nucleolus, as well as in chloroplasts and mitochondria. A central determinant for intracellular distribution of Fe is nicotianamine (NA) generated by NICOTIANAMINE SYNTHASE (NAS). Here, we used *Arabidopsis thaliana* plants with disrupted *NAS* genes to study the accumulation of nucleolar iron and understand its role in nucleolar functions and more specifically in rRNA gene expression. We found that *nas124* triple mutant plants, which contained lower quantities of the iron ligand NA, also contained less iron in the nucleolus. This was concurrent with the expression of normally silenced rRNA genes from nucleolar organizer regions 2 (NOR2). Notably, in *nas234* triple mutant plants, which also contained lower quantities of NA, nucleolar iron and rDNA expression were not affected. In contrast, in both *nas124* and *nas234*, specific RNA modifications were differentially regulated in a genotype dependent manner. Taken together, our results highlight the impact of specific NAS activities in RNA gene expression. We discuss the interplay between NA and nucleolar iron with rDNA functional organization and RNA methylation.

## Teaser

Low iron in the nucleolus and RNA modification changes suggest a central role of nicotianamine synthesis in rRNA gene expression.

## Introduction

The nucleolus is a multifunctional structure linked to ribosome biogenesis, assembly of ribonucleoprotein complexes, nuclear chromatin organization, sequestering of proteins, and stress response (Boulon *et al.*, 2010; Hernandez-Verdun *et al.*, 2010; Stepinski, 2014; Tsai and Pederson, 2014; Pederson and Powell, 2015; Tsekrekou *et al.*, 2017).

The plant nucleolus has an additional, and less investigated, role as a pool of iron and for the intracellular distribution of that iron (Roschzttardtz *et al.*, 2011). Iron is one of the most important micronutrients in plants, implicated in almost every cellular process. Iron excess or deprivation lead to severe growth phenotypes in plants, decrease in biomass production, and chlorosis of leaves (Briat *et al.*, 2015). Accumulation of iron in the nucleolus is intriguing also from the perspective that several neurodegenerative diseases are related to nucleolar iron in human cells (Quintana *et al.*, 2006; Heeney and Finberg, 2014; Kumar *et al.*, 2016). In Alzheimer’s disease, iron damages DNA (Henle *et al.*, 1996, 1999) and binds directly to ribosomal RNA (rRNA), as well as ribosomes, leading to their oxidation and impacting translation efficiency (Honda *et al.*, 2005). Despite these deleterious effects of nucleolar iron, recent studies point out a regulatory role of iron on ribosome assembly and function under specific cellular conditions. The ribosome is particularly dependent on Mg^2+^ to fold and maintain stability due to the negative charge of the rRNA backbone. However, ribosomal Mg^2+^ can be substituted with other divalent cations, including Fe^2+^ and the ribosome can competently mediate translation in this state (Bray *et al.*, 2018; Smethurst *et al.*, 2020; Smethurst and Shcherbik, 2021). Notably, while Fe^2+^ interactions with the ribosome were relevant in ancient ribosomes (containing Mg^+2^ before the evolution of photosynthesis and the increase in molecular oxygen), little is known how iron impact nucleolus organization and ribosome synthesis in present-day oxidative environmental conditions.

**Fig. 1.**
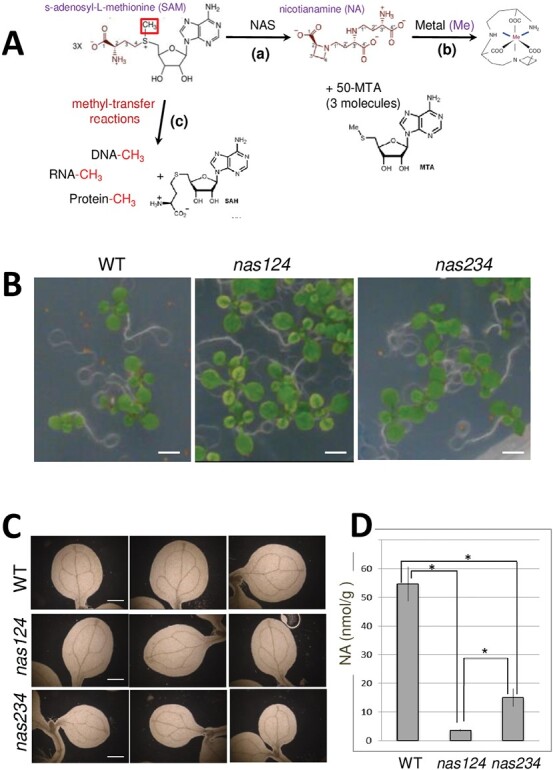
Nicotianamine synthesis and *nas124* and *nas234* plants. (A) The synthesis of NA by NAS from three molecules of SAM includes two carboxypropyl group transfers and one azetidine ring formation, with three molecules of 50-methylthioadenisine (50-MTA) released. NA chelates metal ions including iron. SAM is also a methyl donor in methylation reactions including methylation of DNA, RNA, and proteins. (B) WT, *nas124*, and *nas234* plants grown for 12 d *in vitro*. Bar=2.5 mm. (C) Cleared cotyledon leaves of WT and *nas124* and *nas234* plants, viewed under dark-field illumination. Scale bar=0.4 mm. (D) Nicotianamine (NA) content in 15-day-old *in vitro* grown WT, *nas124*, and *nas234* plants. Data are means ±SE of n=3 independent pools of three plants each, * indicates statistical difference with a Wilcoxon test, *P*<0.1.

Nicotianamine (NA) is a key ligand for essential metals, including iron (Curie and Briat, 2003). NA is an amino acid derivative, synthesized from three S-adenosyl-methionine (SAM) moieties by NICOTIANAMINE SYNTHASE (NAS) [Fig. 1A and references in (Klatte *et al.*, 2009)]. The genome of *A. thaliana* contains four *NAS* genes (Bauer *et al.*, 2004). In the quadruple *nas* mutant (*nas4x-2*), NA is not detectable in rosette leaves; *nas4x-2* also shows a chloronerva-like phenotype and is sterile (Klatte *et al.*, 2009).

Here, we demonstrate that in specific Arabidopsis *nas* mutant plants the nucleolar localization of iron is inhibited. Concomitantly, in these *nas* mutants, rDNA genes from normally silenced NOR2 are expressed and RNA and methylation profiles changed. We discuss how inhibition of NAS activity might affect rDNA gene expression and/or methylation of RNAs.

## Materials and methods

### Plant material and growth conditions

All lines were derived from *A. thaliana* Columbia-0 ecotype. Seeds corresponding to *nas1-2* (SALK_082174), *nas1-1* (GK223A09), *nas2-2* (SALK_066962), *nas3-2* (SALK_106467), and *nas4-1* (SALK_135507) mutant plants were obtained from the Nottingham Arabidopsis Stock Centre (https://arabidopsis.info/). Crosses between single homozygous T-DNA insertion lines were performed to generate triple homozygous mutants, and were identified by PCR and RT–PCR (Supplementary Fig. S1). The mutant *nuc1-2* was previously described (Pontvianne *et al.*, 2010; Durut *et al.*, 2014).

Plants were grown either in soil for 21 d, or *in vitro* on 1× Murashige and Skoog medium (Duchefa Biochemie M0231), including Gamborg B5 vitamins, supplemented with 0.05% (w/v) 2-(*N*-morpholino)ethanesulfonic acid (MES) and 1% (w/v) plant agar (pH was adjusted to 5.7 with KOH) for 15 d. For the soil-grown plants, only the aerial parts were collected for analysis, whereas under the *in vitro* conditions the whole seedlings were analysed. Plants were grown under an 8 h dark/16 h light photoperiod at 21°C.

### Nicotianamine (NA) analysis

Approximately 0.1 g of fresh 15-day-old *in vitro* plants was ground to a fine powder in liquid nitrogen and analysed for NA amount by reversed-phase high-performance liquid chromatography (RP-HPLC) with pre-column derivatization with *o*-phthalaldialdehyde, as previously described (Klatte *et al.*, 2009).

### Molecular cloning and plasmid constructs

For construction of the Fib2-roGFP plasmid, the GAR domain (amino acids 1–73) of the Arabidopsis Fibrillarin 2 (Fib2) gene was PCR amplified with Phusion polymerase (Finnzymes) using the primers *Fib2_KpnI* and *Fib2_BamH1* to add *Kpn*I and *Bam*HI restriction sites (Supplementary Table S1). The PCR product was cloned blunt-end into the vector pGEM-T (Promega). The GAR domain was then subcloned into the expression vector pBinAR-GRX1-roGFP2 (Marty *et al.*, 2009) with *Kpn*I and *Bam*HI. The construct was transformed into *Agrobacterium* strain C58C1 and used for Arabidopsis transformation.

### Confocal laser-scanning microscopy and redox ratio analysis

Confocal microscopic observations were carried out using an Axio observer Z1 microscope with a LSM 700 scanning module and ZEN 2010 software (Zeiss). Excitation of roGFP2 was performed at 488 and 405 nm and a bandpass (BP) 490–555 nm emission filter was used to collect the roGFP2 signal. For background subtraction, the signal was recovered using a BP 420–480 nm emission filter during excitation at 405 nm. Image analyses and quantifications were performed as previously described (Gutscher *et al.*, 2008), using the public domain image analysis program ImageJ 1.52i (https://imagej.nih.gov/ij/).

### Cleared cotyledons and Perls staining coupled to diaminobenzidine (DAB) intensification

To observe vein patterning, cotyledons from 10-day-old WT, *nas124*, and *nas234 in vitro* seedlings were cleared in a 3:1 (v/v) ethanol:acetic acid solution, rinsed and conserved in 70% ethanol, and observed under dark-field illumination. For Perls/DAB staining, leaf fragments from 15-day-old *in vitro* seedlings were vacuum-infiltrated with a fixation solution containing 2% (w/v) paraformaldehyde, 1% (v/v) glutaraldehyde, 1% (w/v) caffeine in 100 mM phosphate buffer (pH 7). The fixed samples were treated and analysed for Fe histochemical staining according to Roschzttardtz *et al.* (2009). Vein pattern and Pearls/DAB observations were performed using LEICA MZ12 and DFC425 digital microscope camera systems.

### Transmission electron microscopy (TEM)

Roots from 5-, 10-, and 15-day-old plants, *in vitro* grown, were first fixed with 3% (v/v) glutaraldehyde in 0.025 M cacodylate buffer (pH 7.3) at room temperature. After washing, the samples were post-fixed with 1% OsO4 in the same buffer. The samples were then dehydrated in a methanol series (30, 50, 70, and 100%) at room temperature. The samples were acetylated and methylated with a freshly prepared 5:1 (v/v) methanol/acetic anhydride mixture at 25°C. Samples were then washed in pure methanol and embedded in Epon 812 resin (Sigma). Ultrathin sectioning was performed on an ultramicrotome (Leica Ultracut), and counterstained with uranyl acetate and lead citrate before being observed using a 7500 Hitachi TEM.

### Fluorescence *in situ* hybridization (FISH)

The FISH probe containing intergenic spacer (IGS) and 5ʹ external transcribed spacer (5ʹETS) rDNA sequences (-220/+250) was amplified with primers *o112/o113* (Supplementary Table S1) and cloned into a pGEM-T^®^ vector (Promega) and amplified by PCR using universal primers T7/M13R, and supplying biotin-16-dUTP (Roche) to the reaction. The analysis was performed using nuclei from leaves of 21-day-old plants grown in soil. The biotin-labelled probe was detected using digoxigenin (1:200; Roche) followed by a sheep anti-digoxigenin antibody conjugated with the fluorochrome Alexa 480 (1:200; Invitrogen). Slides were prepared using Vectashield (Vector Laboratories) mounting medium supplemented with 1 μg/mL 4ʹ,6-diaminido-2-phenylindole (DAPI) and then observed by confocal microscopy (Zeiss LSM700 scanning microscope).

### DNA and RNA isolation

DNA was extracted from liquid nitrogen ground powder by using a DNeasy^®^ Plant Mini Kit (Qiagen). Alternatively, DNA for bisulfite analysis was extracted with illustra™ Nucleon™ PhytoPure™ (GE Healthcare). For RNA, around 800 µl of frozen powder were supplemented with 5 ml of Tri Reagent^®^ (Molecular Research Center, Inc.), then 1 ml of cold chloroform was added. After a 3 min incubation, the mix was centrifuged at 8000 g for 15 min at 4°C. Then 3 ml of cold isopropanol was added to the conserved aqueous phase and the resulting product was then centrifuged at 8000 g for 30 min at 4°C. Isopropanol was replaced by 75% ethanol and the mix was incubated overnight at –20°C. The pellet was dried and resuspended in 65°C diethylpyrocarbonate (DEPC)-treated water. DNA was removed from samples by using the TURBO DNA-free^TM^ kit (Invitrogen), according to the manufacturer’s instructions.

### PCR, RT–PCR, and qPCR

RT–PCR reactions were performed using SuperSript IV Reverse Transcriptase (ThermoFisher Scientific). For cDNA production we simultaneously used random and oligo dT primers. The protocol was performed following the manufacturer’s instructions. The cDNAs obtained were used for later PCR experiments. PCR amplifications were carried out with GoTaq® DNA Polymerase and 5× GoTaq^®^ Green Buffer (Promega). 3ʹETS products from RT–PCR and PCR reactions were loaded on an agarose gel (2% agarose) in 0.5× Tris-acetate-EDTA (TAE) supplemented with Gel Red^®^ (Interchim, Roche). Quantitative PCR (qPCR) was performed using a LightCycler 480 system and Taykon^TM^ qPCR for SYBR^®^ Assays (Eurogentec) following the manufacturer’s instructions. All primer pairs used in RT–PCR, PCR, and qPCR are provided in Supplementary Table S1.

### Bisulfite DNA treatment and analysis

DNA was converted (me5C to T) using the EpiTect Plus Bisulfite Conversion Kit (Qiagen). The target region was then amplified by PCR using the Ex Taq DNA Polymerase, Hot Start Version Kit (TaKaRa Bio Inc.) and oligo primers *o112/o113* and *o124/o125* (Supplementary Table S1) to amplify promoter/5ʹETS and 3ʹETS, respectively. 3ʹETS PCR products were run on an agarose gel, sliced from the gel, and purified using a GeneClean® Turbo Kit (MP Biomedicals). PCR products were cloned into a pGEM®-T easy vector (Promega), transformed into *Escherichia coli* DH5α, and sequenced using T7 and SP6 primers. The sequences obtained were mapped to a reference sequence and the alignment was sent to CyMATE to analyse methylation sites in the sequence. The results were analysed using the prop.test function in R software to verify significant differences with R software (Pontvianne *et al.*, 2012).

### RiboMethSeq analysis and detection of nucleosides using the multiple reaction monitoring (MRM) method in mass spectrometry

RiboMethSeq analysis was performed using the method described in Marchand *et al.* (2016) and recently used in Arabidopsis plants (Azevedo-Favory *et al.*, 2021).

#### Enzymatic processing of RNA.

 Total RNA (~400 ng) from 15-day-old *in vitro* grown WT, *nas124*, and *nas234* plants was digested with 0.001 U of Nuclease P1 (Sigma, N8630) and 3 µl of 0.1 M ammonium acetate (pH 5.3) for 2 h at 42 °C. Then a dephosphorylation of nucleosides was performed with 0.001 U of alkaline phosphatase (Sigma, P4252) and 3 µl of 1 M ammonium acetate for 2 h at 37°C. Next, the mixture was diluted twice and was filtered with 0.22 µm filters (Millex®-GV, Millipore, SLGVR04NL). Five microlitres of each sample was injected in triplicate into a liquid chromatography coupled to tandem mass spectrometry (LC-MSMS) and then methylated/unmethylated ratios were estimated. Since the adenosine signal was saturated in the samples, we used uridine (U) to estimate ratios.

### RNA-seq and bioinformatics analyses

Total RNA from either 15-day-old *in vitro* grown WT or *nas124* mutant plants was prepared to generate three biological replicates per sample. Sequencing was performed by the Plateforme transcriptOmique de l'iPS2 (POPS) Facility (Institut de Génomique-CNS, Evry, France) from stranded ribozero RNA-seq libraries and using NextSeq500 (Illumina) and Hiseq2000 to generate 2 × 75-bp-long reads. Raw reads were trimmed using Trimmomatic v0.39 (Bolger *et al.*, 2014). Trimmed reads corresponding to mitochondrial, chloroplastic, and rRNA sequences were filtered out using bowtie2 v2.3.5 (Langmead and Salzberg, 2012) in ‘sensitive-local’ mode. Read mapping against the TAIR10 genome with the Araport11 gtf file was performed using Hisat2 v2.1.0 (Kim *et al.*, 2015). Read counting was performed using htseq-count v0.12.4 (Anders *et al.*, 2015) in ‘union’ mode and normalized by total of mapped reads (reads per millions, rpm). Differential analysis was performed using the Bioconductor R v4.1.2 package DESeq2 (Love *et al.*, 2014) with a false discovery rate of 0.05. P values were corrected for multiple tests by the Benjamini–Hochberg rule (adjusted P value). The up-regulated genes were defined with a fold change minimum to 2 and the down-regulated genes with a fold change less than 1. The heat map was obtained using the heatmap.2 function of the gplots R package (https://CRAN.R-project.org/package=gplots) using Pearson distance and an average method for the hierarchical clustering. To identify differentially expressed long non-coding RNAs (DE lncRNAs), Col-0, and *nas124*, Illumina reads were aligned to the Arabidopsis Transcriptome ReconstructoR pipeline (Ivanov *et al*., 2021) All WT and *nas124* sequences were submitted to the Sequence Read Archive (SRA): PRJNA782822 (https://dataview.ncbi.nlm.nih.gov/object/PRJNA782822?reviewer=ee044uijk32bst5p1v52bogou9). Gene Ontology (GO) analysis was performed using PANTHER (http://www.pantherdb.org/).

### Enzyme activity measurements and detection of reactive oxygen species

To measure enzyme activity, plantlets (~200 mg) were harvested, immediately frozen in liquid nitrogen, and conserved at -80°C until assay. Plant material was ground in liquid nitrogen (CryoMill, Retsch), resuspended in 400 µl of cold buffer (phosphate buffer 50 mM pH 7,2, 1 mM EDTA, 2% of polyvinyl pyrrolidone, 1 tablet of complete protease inhibitor cocktail (Roche) for 10 ml) before high-speed centrifugation (21000 g, 4°C, 10 min). The supernatant was conserved on ice until enzyme analysis (Veljovic-Jovanovic *et al.*, 2001). CAT (catalase) activity was measured according to Cakmak and Marschner (1992). The assay depends on the decrease in absorbance at 240 nm as H_2_O_2_ is degraded. The reaction mixture 1 ml containing 25 mM sodium phosphate buffer (pH 7.0), 10 mM H_2_O_2_ and 0.1 ml enzyme extract. The reaction was started by adding H_2_O_2_. APX (ascorbate peroxidase) activity was measured according to Nakano and Asada (1981). The assay depends on the decrease in absorbance at 290 nm as ascorbate is oxidized. The 1 ml reaction mixture contained 25 mM sodium phosphate buffer (pH 7.0), 0.5 mM ascorbate, 0.1 mM H_2_O_2_, 0.1 mM EDTA and 0.1 ml enzyme extract. The reaction was started by adding H_2_O_2_.

Detection of ROS was performed as previously described (Mhamdi *et al.*, 2010). Staining was performed at room temperature on Col-0 and *nas124* 7-day-old plants. For the detection of H_2_O_2_, plants were vacuum infiltrated in the nitro blue tetrazolium (NBT) staining medium [10 mM phosphate buffer pH 7.5, 10 mM Na azide, 1 tablet of NBT (10 mg Sigma)]. For the detection of superoxide, plants were vacuum infiltrated in 5 mM DAB at pH 3.8. For both staining, plants were then incubated in the same medium until the coloration was observed and then chlorophyll was removed in 95% ethanol before pictures taking.

### Primer extension and northern blot analysis

Total RNA extractions and 5' end-labelling of oligo probes (p23, p43, p5 and p6, tis, p, u3, p18S and p25S; see Supplementary Table S1) were performed as described previously (Pontvianne *et al.*, 2010). Northern blots and primer extension gels were performed using respectively 3 μg and 15 μg of total RNA from 21-day-old plants grown in soil. Two northern blot membranes were pre-incubated in PerfectHyb Plus hybridization buffer (Sigma) for at least 3 h at 42 °C. Labelled probes p5, p23, p43, p18S and p25S were then added (1µl at 10µM) and incubated overnight at 42 °C. Membranes were washed at 50 °C during 15 min with 2X SSC 0.1% SDS, then with 0.5X SSC, 0.1% SDS, and finally with 0.1X SSC 0.1% SDS. Primer extension and dideoxy sequencing reactions were performed according to Pontvianne *et al.* (2010). Northern blots and primer extension reactions were analysed on a Personal Molecular Imager (PMI, BioRad) and quantified using Quantity One software.

## Results

### Plant phenotype and NA accumulation in *nas124* and *nas234* plants

The genome of *A. thaliana* contained four *NAS* genes that were differentially expressed. *NAS1*, *NAS2*, and *NAS4* were essentially expressed in roots, whereas *NAS3* was expressed at low levels in roots and leaves. Single T-DNA insertion lines did not show any visible phenotype (Bauer *et al.*, 2004) and had WT-nicotianamine levels (Klatte *et al.*, 2009). We first characterized the *nas124* and *nas234* triple mutant plants obtained from single *nas1-2* (SALK_082174), *nas2-2* (SALK_066962), *nas3-2* (SALK_106467), and *nas4-1* (SALK_135507) mutant plants (Supplementary Fig. S1A, B).

The *nas124* and *nas234* triple mutants grown in soil did not show growth and/or plant developmental defects compared to the WT plants, though the first leaves from *in vitro* grown *nas124* displayed a chloronerva-like phenotype (Fig. 1B). Cleared cotyledons from *nas124* and *nas234* showed disturbed vein patterns (Fig. 1C). Furthermore, *nas124* and *nas234* contained 6.5% and 27%, respectively, of the NA concentration compared to the WT (Fig. 1D). It was expected that residual NA detected in *nas124* came from low levels of NAS3 expressed in roots and leaves, whereas NA detected in *nas234* came from highly expressed NAS1 in roots compared to leaves (Supplementary Fig. S1C).

### Loss of nucleolar iron in *nas124*

**Fig. 2.**
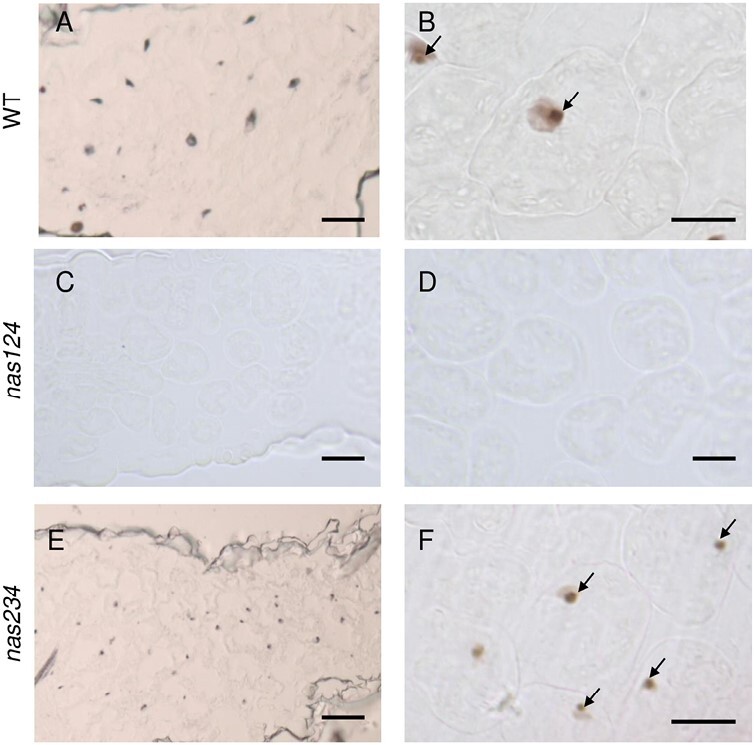
Perls/DAB staining to detect nucleolar iron. Nucleolar iron in *nas124* and *nas234* leaf sections showing mesophyll cells from 15-day-old WT (A and B), *nas124* (C and D), and *nas234* (E and F) plants grown *in vitro* were stained with Perls/DAB. Arrows indicate nucleolar iron in WT and *nas234*. Scale bars=40 µm for A, C, and E, and 20 µm for B, D, and F.

NA is a central ligand for intracellular iron (Bauer *et al.*, 2004; Klatte *et al.*, 2009). Therefore, we determined the impact of reduced NA content on the accumulation of nucleolar iron in *nas124* and *nas234* plants. We performed Perls associated with diaminobenzidine (Perls/DAB) staining (Fig. 2; Supplementary Fig. S2A). The Perls/DAB staining detected iron in the nucleolus of WT and *nas234* mesophyll cells (black staining, upper and lower panels). However, no iron was detected in *nas124* nucleoli (middle panel; Fig. 2). Similarly, Perls/DAB staining of roots cells detected iron in WT and *nas234* nucleoli but not in *nas124* nucleoli (Supplementary Fig. S3). We also observed that iron formed aggregates in the xylem vessels of *nas124* that might correspond to iron-ferritin complexes or Fe precipitates of unknown origin (Supplementary Fig. S2). In addition, increased ferritin protein levels were detected in *nas124* (Supplementary Fig. S2B). Altogether these results show that simultaneous disruption of *NAS1*, *NAS2*, and *NAS4* genes, and decreased NA amount provoked loss of nucleolar iron.

### Absence of iron in *nas124* nucleoli does not change nucleolar/nuclear redox state

**Fig. 3.**
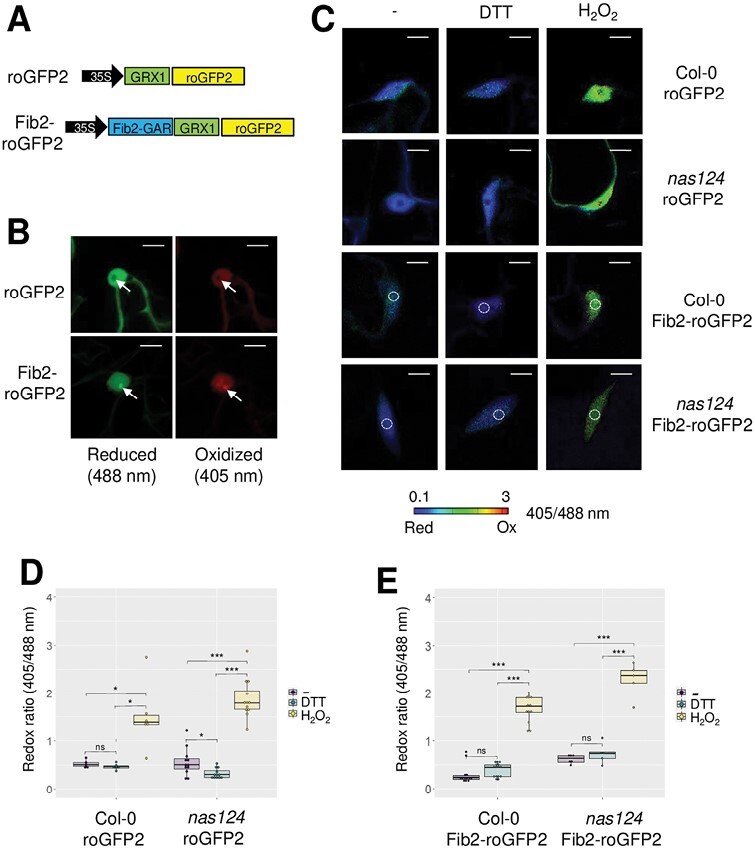
*In vivo* monitoring of the nucleolar/nuclear redox state in *nas124*. (A) Gene structure of the roGFP2 constructs used to transform WT and *nas124* plants. Both constructs were under the control of the 35S-CaMV promoter. In Fib2-roGFP2, GRX1 was fused to roGFP2 as in roGFP2, but the GAR domain (1–73 N-terminal amino acids) of Fib2 was additionally fused to GRX1-roGFP2. (B) Confocal images of nuclei of WT cotyledons expressing roGFP2 or Fib2-roGFP2. RoGFP2 was excited at 488 nm or 405 nm to monitor the reduced or oxidized forms, respectively. Arrows indicate the nucleolus. Scale bars=10 µm. (C) Steady state ratio images of WT or *nas124* plants expressing roGFP2 or Fib2-roGFP2 and calculated as the 405/488 nm fluorescence. To fully reduce or oxidize the sensor, seedlings were immersed in 10 mM DTT or 100 mM H_2_O_2_, respectively. Control samples were immersed in MS/2 liquid medium only. False colours indicate the fluorescence ratio on a scale from blue (reduced) to red (oxidized). Scale bars=10 µm. White circles indicate the location of the nucleolus. (D-E) Fluorescence ratio of roGFP2 (D) and Fib2-roGFP2 (E) calculated from ratio images. Ratios were calculated from six to 16 nuclei per sample. Asterisks indicate a significant difference calculated by Student’s *t*-test (* *P*≤0.05, ** *P*≤0.01, and *** *P*≤0.001, ns *P*>0.05).

As the ferrous ion (Fe^2+^) is highly reactive with H_2_O_2_ to produce hydroxide (OH^-^) and hydroxyl (OH^.^) radicals through Fenton reactions, we reasoned that the contrasted iron accumulation in the nucleolus between WT and *nas124* plants might change the redox state of the nucleolus or the surrounding nucleoplasm. To monitor the nuclear redox state in WT and *nas124* plants, we used the redox sensor roGFP2 (Fig. 3A) in which the roGFP2 is fused to the glutaredoxin GRX1 (Gutscher *et al.*, 2008) and indicates the redox state by excitation at different wavelengths. This construct is expressed in the cytosol and the nuclear compartments but is excluded from the nucleolus (Fig. 3B). To also monitor the redox state in the nucleolus, we fused GRX1-roGFP2 with the GAR domain of the Fibrillarin2 (Fib2) protein which targets the protein to the nucleolus (Barneche *et al.*, 2000) and documents the redox state in the nucleolus and in the nucleoplasm (Fig. 3B). The system was calibrated with H_2_O_2_ and DTT, respectively, to fully oxidize or reduce roGFP2 and Fib2-roGFP2, and confirmed that both sensors were similarly responsive to the redox environment (Fig. 3C-E). When comparing the fluorescence ratio (405/488 nm) in WT and *nas124* plants, we observed that the ratio was close to the ratio measured under DTT treatment, suggesting that in both genetic backgrounds the redox sensor is fully reduced in the nucleolus and the nucleus (Fig. 3D-E). Therefore, we concluded that different iron contents in WT and *nas124* nucleoli do not impact the nucleolar or the nuclear redox state.

Furthermore, we measured reactive oxygen species (ROS) accumulation *in planta* by staining *nas124* plants with 3,3ʹ-diaminobenzidine (DAB) and nitro blue tetrazolium (NBT) that react with H_2_O_2_ and O_2_^.-^, respectively. Similar staining was found in *nas124* and WT plants (Supplementary Fig. S4A). Major ROS detoxification enzymes, catalases, and ascorbate peroxidases also had similar activities in WT and *nas124* plants (Supplementary Fig. S4B). In summary, *nas124* mutants did not experience oxidative stress due to perturbed iron distribution.

### Transcriptome analysis of *nas124* plants

**Fig. 4.**
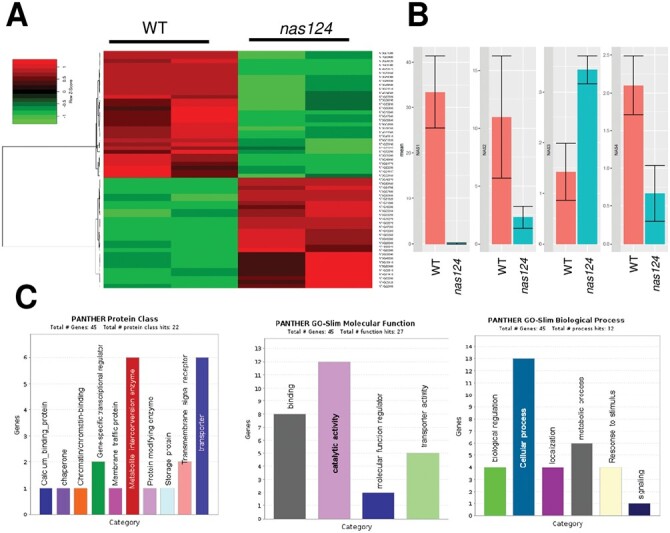
Transcriptomic analysis of *nas124*. (A) Heat map of 60 DE genes in 15-day-old *in vitro* grown *nas124* compared to WT plants. The colour key, histogram, and values are shown. Down- and up-regulated genes are in green and red, respectively. (B) Graph of normalized reads (rpkm) in WT and *nas124* plants for *NAS1* (At5g04950), *NAS2* (At5g56080), *NAS3* (At1g09240), and *NAS4* (At1g09240) transcripts, respectively, from left to right on the graph. Graphs and plots were generated using RStudio, Ri 386 version 4.1.1. (C) GO analysis for protein classes, -Slim molecular function, and -Slim biological process.

Disruption of nucleolar organization affects nuclear gene expression in *A. thaliana* (Pontvianne *et al.*, 2016). To investigate the impact of reduced nucleolar iron and/or decreased NA on global gene expression, we performed RNAseq analysis of WT and *nas124* plants, with good correlation between two replicates (Fig. 4; Supplementary Fig. S5A; Supplementary Table S2A). We identified 60 differentially expressed (DE) genes with a false discovery rate (FDR) lower than 5%. Among them were 28 up-regulated genes with fold-change (log2) values (*nas124*/WT) ≥2, and 32 down-regulated genes with fold-change (log2) values ≤0.5 (Fig. 4A; Supplementary Table S2B). The most down-regulated gene was *NAS1* (–8.1-fold) and the most up-regulated gene encoded for the hypothetical protein At1g64795 (8.1-fold). The *NAS2* and *NAS4* genes did not appear among the 60 DE genes, nevertheless a decreased number of *NAS2* and *NAS4* reads (–2.2- and –1.6-fold, respectively) and a slightly increased number of NAS3 reads (1.3-fold) were observed in *nas124* plants (Fig. 4B; Supplementary Table S2). Down-regulated genes other than *NAS* were the iron metabolism-related gene *FERRETIN 1* (At5g01600) and defence or pathogen response genes, including the S-adenosyl-l-methionine-dependent methyltransferase superfamily protein gene (At2g32160). Among the detected up-regulated genes were *OLIGOPEPTIDE TRANSPORTER*, *OPT3* (At4g16370), *IRON REGULATED TRANSPORTER 3*, *IRT3* (At1g60960), and *IRONMAN 2* (At1g47395) (Supplementary Table S2B). Furthermore, a GO (GO-PANTHER) analysis of protein classes highlighted two categories: ‘metabolism interconversion enzymes’ (six DE genes) and ‘transporters’ (six DE genes), whereas the GO-Slim molecular function and biological processes highlighted the categories ‘catalytic activity’ (12 DE genes), ‘binding’ (eight DE genes), and ‘cellular processes’ (13 DE genes) (Fig. 4C; Supplementary Table S3). Lastly, further analysis to determine de-regulation of non-coding RNA did not reveal significant transcriptomic variations of small RNAs (snoRNA, snRNA, tRNA) or long non-coding RNAs in *nas124* plants (Supplementary Fig. S5B). Taken together, RNAseq analysis shows that simultaneous disruption of *NAS1*, *NAS2*, and *NAS4* affects expression of genes involved in metal metabolism and/or transport, and accumulation of transcripts associated with plant defence or pathogen response.

### Functional organization of *nas124* nucleoli

**Fig. 5.**
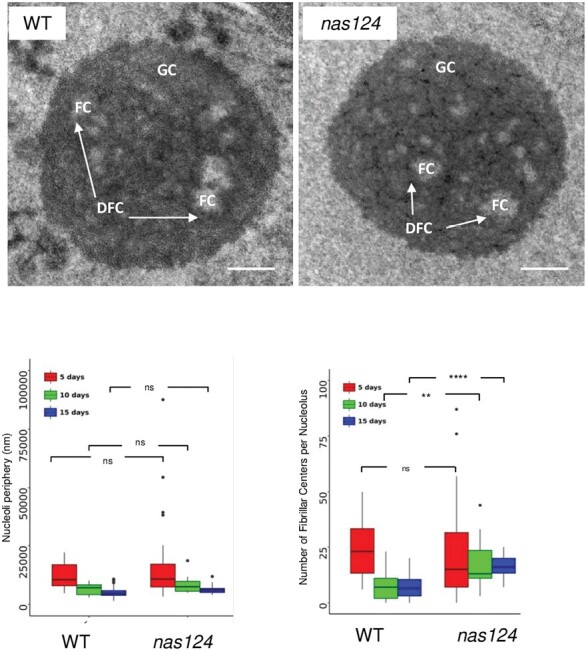
Nucleolus organization in *nas124*. Top, TEM images of root cells from 10-day-old WT and *nas124* plants grown *in vitro*. The Fibrillar Centers (FC) are surrounded by the Dense Fibrillar Component (DFC, indicated by white arrows) and embedded in the Granular Component (GC). Scale bar=500 nm. Bottom, boxplot graphs of nucleoli periphery (left) and number of FC structures per nucleolus (tight) in 5-, 10-, and 15-day-old root cells. The dots indicate the outlier samples. 28 (5-day-old), 13 (10-day-old), and 46 (15-day-old) WT nucleoli and 35 (5-day-old), 19 (10-day-old), and 16 (15-day-old) *nas124* nucleoli were analysed. Asterisks indicate a significant difference calculated by Student’s *t*-test: ns *P*>0.05, ** *P*≤0.01, and **** *P*≤0.001.

We carried out observations by TEM to investigate if reduced amounts of nucleolar iron in *nas124* plants could impact nucleolar structural and/or functional organization (Fig. 5). In the nucleolus, three major functional structures are observed: fibrillar centres (FC), where transcriptionally active 45S rDNA localizes, dense fibrillar components (DFC), the place of primary rRNA processing events, and the granular component (GC), mainly composed of pre-ribosome particles. Transcription of rDNA by RNA Pol I occurs at the interface of the FC and the DFC.

In plant cells, there are two types of FCs: homogeneous FCs, small, numerous, and associated with active nucleoli, and heterogeneous FCs, fewer and larger, associated with low rates of nucleolar activity (Saez-Vasquez and Medina, 2008). To address this issue in *nas124*, we performed TEM of nucleoli from 5-, 10-, and 15-day-old WT and *nas124* plants. Then we measured nucleoli periphery and determined the number of FC per nucleolus (left and right graphs respectively in Fig. 5). In 5-day-old plants, nucleoli from both genotypes were larger than nucleoli from 10- and 15-day-old plants (Fig. 5). Also 5-day-old plant nucleoli from WT and *nas124* were similar in size and shape, containing between 5 and 30 FC. In 10-day-old plants, WT and *nas124* nucleoli also had similar size and functional (FC, DFC, and GC) organization. However, *nas124* nucleoli contained around twice the number of FC structures than WT nucleoli. Likewise, nucleoli from 15-day-old WT and *nas124* plants displayed similar sizes, but a higher number of FC was observed in *nas124* (between 12 and 30 FC) than in WT (≤15 FC). Taken together, the TEM results suggested that *nas124* nucleoli were more active compared to WT nucleoli.

### NOR organization and expression in *nas124*

The genome of *A. thaliana* Col-0 contains around 1500 copies of 45S rDNA units per diploid genome (Saez-Vasquez and Gadal, 2010; Layat *et al.*, 2012; Mohannath *et al.*, 2016). They localize in the nucleolar organizer regions (NORs) from chromosomes 2 and 4 (NOR2 and NOR4) (Copenhaver and Pikaard, 1996a, b); however, only rDNA (VAR2 and 3) from NOR4 is transcribed in most plant organs. The rDNA (VAR1) from NOR2 is repressed by epigenetic mechanisms and expressed mainly early during seed germination and in specific mutant plants with nucleolar disorganization (Pontvianne *et al.*, 2010), lower copy numbers (Pontvianne *et al.*, 2013), or altered proportion of rDNA variants (Durut *et al.*, 2014).

**Fig. 6.**
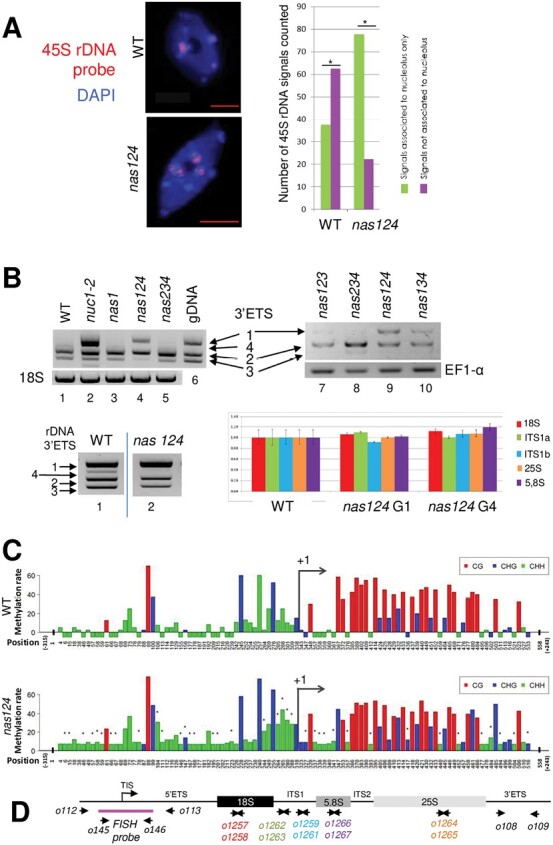
NOR expression in *nas124*. (A) FISH analysis of 45S rDNA performed on leaf cells from 21-day-old WT and *nas124* plants grown in soil. 45S-labelled probes hybridized to NOR2 and NOR4 (red signals). DNA in the nucleoplasm is stained in blue with DAPI and the black area in the nucleus corresponds to the nucleolus that is not stained because the rDNA chromatin is decondensed and too thin to be stained by DAPI (Pontvianne *et al.*, 2016a). Scale bars=5 µm. The histogram shows the number of 45S FISH signals in 32 WT and 18 *nas124* cells. (B) Top panels, RT–PCR to detect 45S pre-rRNA variants in leaves from 21-day-old WT (lane 1), *nuc1-2* (lane 2), *nas1* (lane 3), *nas124* (lanes 4 and 9), *nas234* (lanes 5 and 8), *nas123* (lane 7), and *nas134* (lane 10) plants grown in soil. Amplification of 18S (lanes 1–5) and EF1-alpha (lanes 7–10) RNA transcripts was performed to verify the similar amount of RNA in each sample. Amplification of genomic DNA (lane 6) shows up to four rDNA variants in *A. thaliana* Col-0 plants. Bottom panels, PCR (left) analysis of 3ʹETS rDNA in WT (lane 1) and *nas124* (lane 2) plants and RT–qPCR (right) of 18S, Internal Transcribed Spacer 1 (ITS1a and ITS1b), 25S and 5.8S rDNA sequences in WT and the *nas124* siblings (G1 and G4). (C) Bisulfite analysis of promoter/5ʹETS (from –315 to +243) rDNA sequences from WT and *nas124* plants. The graph bars show CG, CHG, and CHH methylation levels at different positions in the rDNA. The tiny dark stars over the graph bars indicate sites with significant (*P* ≤0.05) difference of methylation rate between WT and *nas124* for each position. (D) The schematic of a 45S rDNA unit shows the position of primers used in bisulfite sequencing analysis (o112/o113) to amplify rDNA for the FISH probe (o145/o146) to detect 3ʹETS (o108/o109) by PCR, and 18S (o1257/o1258), ITS1 (o1262/o1263 and o1259/o1261), 5.8S (o1266/o1267), and 25S (o1264/o1265) by qPCR.

Active NORs are associated to the nucleolus while inactive NORs remain at the nuclear periphery (Sáez-Vásquez and Delseny, 2019). To examine nucleolus association/dissociation of NORs in *nas124* plants, we performed FISH with a probe homologous to the 45S rDNA (Fig. 6A). In WT nuclei, the FISH probe detected a single and strong signal (active NOR4) associated with the nucleolus, whereas two weak signals close to the nuclear border reflected the non-associated, inactive NOR2. In contrast, in *nas124* nuclei, the FISH probe detected three to four strong signals associated with the nucleolus. The analysis based on 32 WT and 18 *nas124* nuclei revealed around 2-fold more nucleolus-associated and around 3-fold less non-associated signals in *nas124* nuclei compared to WT. This increased association between the nucleolus and NOR2 indicates transcriptional activation of this usually silent rDNA locus.

To verify expression of NOR2 and NOR4 in *nas124* plants, we performed RT–PCR analysis using specific primers that amplify the 3ʹ external transcribed spacer (3ʹETS) rDNA from NOR2 (*VAR1*) and/or from NOR4 (*VAR2* and *3*) (Pontvianne *et al.*, 2010). RT–PCR amplification of the 3ʹETS generated specific product sizes for each rRNA gene variant (Fig. 6B) and confirmed expression of VAR1 in *nuc1-2* plants with disrupted nucleolin 1 (NUC1), as reported (Pontvianne *et al.*, 2010; Durut *et al.*, 2014) and used as a positive control here. The analysis detected expression of rRNA *VAR1* in *nas124*, but not in WT or *nas234* plants. In *nas124* plants, rRNA *VAR2* expression was unaltered, whereas *VAR3* was at same point repressed. We also detected, but at lower level, expression of rRNA *VAR1* in *nas123* and *nas134*. Finally, rRNA *VAR1* was not expressed in *nas1* plants indicating that disruption of *NAS1* alone was not sufficient to induce expression of NOR2.

Furthermore, to determine rDNA copy number variations and/or organization that could have affected expression of rDNA variants, we performed PCR and qPCR using genomic DNA from WT and *nas124*, including a fourth generation of amplified homozygous mutants (Fig. 6B). Except for a lower PCR signal corresponding to rDNA VAR4 observed in *nas124* compared to WT, this analysis shows no obvious differences in the ratio of rDNA variants VAR1-3 in *nas124* compared to WT plants. Similarly, RT–qPCR of ITS1 (Internal Transcribed Spacer 1), 18S, 5.8S, and 25S rDNA sequences excluded significant differences in the total copy number of rDNA in *nas124*, from the 1st to the 4th generation.

Transcriptionally active rDNA is hypomethylated and associated with acetylated histones (H3Ac and H4Ac) or dimethylated histone H3 at lysine 4 (H3K4me2), whereas inactive rDNA is hypermethylated and associated with dimethylated histone H3 at lysine 9 (H3K9me2) (Probst *et al.*, 2004; Espada *et al.*, 2007; Earley *et al.*, 2010). Therefore, we analysed the epigenetic state of 45S rDNA in *nas124* plants. We performed bisulfite analysis of promoter and 5ʹETS rDNA (–315 to +243) sequences (Fig. 6C). Notably, CG sites were more present downstream of Transcription Initiation Site at +1 (TIS/+1), whereas CHG and CHH sites were enriched upstream of TIS/+1. Bisulfite analysis revealed a slight hypermethylation in *nas124* at specific CHG and CHH sites in the 5ʹETS, whereas the CG methylation in *nas124* remained similar to WT. Notably, in the promoter sequence, there was higher CHG and CHH methylation in *nas124* plants compared to WT. Silencing of 45S rRNA genes involves long non-coding RNA (Schmitz *et al.*, 2010) or small interfering RNA (siRNA) that direct specific DNA methylation (Costa-Nunes *et al.*, 2010). Northern blot experiments did not reveal significant differences in the accumulation of promoter siRNA 45S and siR759, or IGS rRNA sequences (Supplementary Fig. S6).

To determine if the CHG and CHH hypermethylation was specific for either *VAR1*, and/or *VAR2*, and/or *VAR3*, we performed bisulfite analysis of the 3ʹETS rDNA region (Supplementary Fig. S7). In contrast to the promoter and 5ʹETS regions, CG, CHG, and CHH sites were more equally distributed in the 3ʹETS of all rDNA variants. The bisulfite analysis did not reveal significant differences in the methylation state between rDNA variants from *nas124* and WT plants. Only one CG site at position 44 in *VAR1* was hypomethylated in *nas124*.

We also examined histone marks by Chromatin Immuno-Precipitation (ChIP) followed by qPCR analysis (Supplementary Fig. S8). Primers matching mature 25S and non-coding (IGS, 5ʹETS, and 3ʹETS) rDNA sequences did not detect changes of either active (H3Ac and H3K4me2) or inactive (H3K9me2) histone marks in *nas124* compared to WT plants.

Together these analyses revealed that among the epigenetic parameters investigated, changes in DNA methylation at rDNA promoter sequences are most evident with expression of rDNA from NOR2 in the *nas124* mutant. In contrast, in the *nas234* mutant we do not observe NOR2 activity and/or nucleolus association or epigenetic changes (Supplementary Fig. S9).

### RNA methylation in *nas124* and *nas234* plants

rRNA is modified concomitantly or immediately after RNA pol I transcription. Reported rRNA modifications include sugar and base methylation and/or acetylation, and serve to stabilize structures of the rRNA scaffold and ensure efficiency and accuracy of translation (Sharma and Lafontaine, 2015; Sloan *et al.*, 2017). Since S-adenosyl-methionine (SAM) moieties used by NAS enzymes to synthesise NA are also donors in methylation reactions, including methylation of DNA, RNA, and proteins (Fig. 1), we questioned whether reduced NAS activity in the *nas124* and *nas234* plants might impact availability of SAM and subsequently methylation of coding and non-coding RNAs. Here, we studied the impact of NAS reduction (in *nas124* and *nas234*) and/or the expression of rRNA from NOR2 (in *nas124*) on RNA base modifications and on 2ʹ-*O*-methylation (2ʹ-*O*-Me), the most abundant rRNA modification (Azevedo-Favory *et al.*, 2021; Wu *et al.*, 2021).

**Fig. 7.**
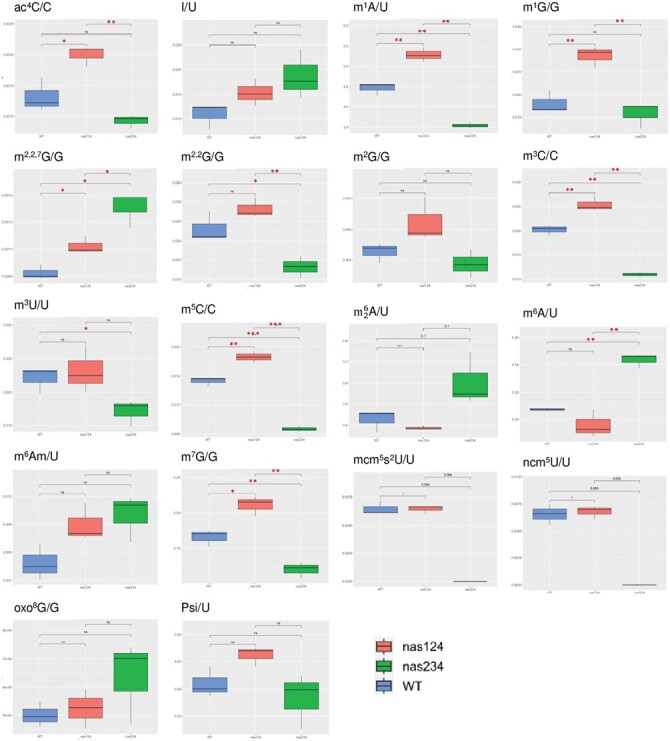
RNA modifications in *nas124* and *nas234*. Graphs show ratio of ac^4^C/C, I/U, m^1^A/U, m^1^G/G, m^2,2,7^G/G, m^2,2^G/G, m^2^G/G, m^3^C/C, m^3^U/U, m^5^C/C, $m_{2}^{6}$A/U, m^6^A/U, m^6^Am/U, m^7^G/G, mcm^5^s^2^U/U, ncm^5^U/U, oxo^8^G/G, and Psi/U detected by the LC-MRM method in WT and *nas124*. *P* values are indicated for each methylated/non-methylated nucleotide ratio. Asterisks indicate a significant difference calculated by Student’s *t*-test: ns >0.05, * *P*≤0.05, ** *P*≤0.01, and ***P≤0.001. Statistical differences with Wilcoxon test, *P*≤0.1 are provided for m^6,6^A/U, mcm^5^s^2^U/U, ncm^5^U/U.

We performed the MRM methodology in mass spectrometry to analyse 18 different RNA base modifications (Fig. 7; Supplementary Fig. S10), including m^1^A, m^5^C, m^7^G, m^3^A, and ac^4^C identified in rRNA from yeast and animal cells (Sharma and Lafontaine, 2015; Sloan *et al.*, 2017). The foremost observation was a significant (≤0.05) increase of methylation of m^1^A, m^1^G, m^2,7,7^G, m^3^C, m^5^C, m^7^G, and also ac^4^C in *nas124*. No decreased methylation of specific RNA modifications was observed in *nas124*. In contrast, methylation of m^1^A, m^3^C, m^5^C, m^7^G, m^2,2^G, m^3^U, mcm^5^s^2^U/U, and ncm^5^U/U was decreased in *nas234*, whereas methylation of m^2,7,7^G and m^6^A increased. No significant changes were detected for I, m^2^G, m^6^Am, $m_{2}^{6}$A, oxo^8^G, and/or pseudouridylation (Psi) in *nas124* or *nas234* (Fig. 7; Supplementary Table S4).

We performed RiboMethSeq analysis to determine 2ʹ-*O*-Me profiles of 18S, 5.8S, and 25S rRNA in *nas124* and *nas234* compared to WT plants. No significant changes were detected in *nas124* or *nas234* plants (Supplementary Fig. S10A; Supplementary Table S5).

Together, these analyses revealed specific RNA base methylation changes in *nas124* and *nas234*. Notably RNA base methylation changes detected in *nas124* were opposite to, or are not affected in, *nas234*.

## Discussion

The *nas124* and *nas234* plants described here each had a reduced concentration of NA; however, specific cellular and molecular features were detected in these mutants. Under standard growth conditions, a reduced concentration of NA did not have an evident impact on *nas124* or *nas234* plant growth or development phenotypes, except for mildly disturbed vein patterns in cotyledons in both genotypes and chloronerva-like phenotypes in primary leaves in *nas124* (Fig. 1). Leaf chlorosis was also observed in the fertile and quadruple mutant *nas4x-1*. However, this chlorosis was minor at the vegetative stage and intensified at the reproductive stage (Klatte *et al.*, 2009).

Nevertheless, the most outstanding observation was that only *nas124* contains low or undetectable iron in the nucleolus (Fig. 2; Supplementary Figs S2, S3). We cannot yet explain the chloronerva-like and low nucleolar iron phenotypes in *nas124*, which do not occur in *nas234* plants. The lower amount of total NA in *nas124* and the lack of NAS activity in roots might have an impact in iron transport from tissue to tissue, cell to cell, and at last to the nucleolus. However, inhibition of iron uptake or increased iron availability did not affect *nas124* growth (root phenotype) compared to WT plants; neither media composition change (MS and/or sugar) affected chlorosis in *nas124* (Supplementary Fig. S11).

Transcriptome analysis did not explain the phenotypes of the *nas124* plants (Fig. 4; Supplementary Fig. S5). However, mRNA transcripts encoding proteins involved in iron metabolism or transport were de-regulated in *nas124* plants, indicating a disturbed sensing of iron availability status in these plants. In addition, variations of transcripts associated with plant defence or pathogen response indicate that *nas124* might be also sensitive to biotic stresses. This is in agreement with the reported role of iron in the plant pathogenesis response (reviewed in Liu *et al.*, 2021).

How reduced NA inhibits nucleolar accumulation of iron in *nas124*, but not in *nas234*, remains an open question. However, the finding of low or undetectable iron in the nucleolus in *nas124* allowed us to address the role of nucleolar iron in plants.

The functional and structural organization of the nucleolus is linked to ribosome biogenesis (Saez-Vasquez and Medina, 2008; Boulon *et al.*, 2010). In *nas124*, the lack of nucleolar iron induces increased number of FC likely due to rRNA VAR1 expression from normally silenced NOR2. It may be expected that rDNA VAR1 expression in *nas124* is a consequence of failed repression rather than activation of NOR2. This is in agreement with steady rDNA *VAR1* expression in *nas124* early during seedling establishment (Supplementary Fig. S12). Indeed, in early states of seedling establishment, NOR2 and NOR4 were both expressed in WT plants. Later, NOR2 becomes progressively silenced (Earley *et al.*, 2010; Benoit *et al.*, 2013; Durut *et al.*, 2014). Expression of NOR2 and low nucleolar iron and the presence of aggregates in the xylem vessels was also observed *nuc1-2* plants (Supplementary Fig. S2). However, in contrast to *nas124*, disruption of NUC1 also induced nucleolus disorganization, chromatin de-condensation, and rDNA hypomethylation (Pontvianne *et al.*, 2007, 2010), indicating that loss of nucleolar iron and NOR2 expression are triggered by different mechanisms.

Expression of rRNA *VAR1* is associated with rDNA CpG hypomethylation, rDNA copy number or ratio, rDNA chromatin decondensation, and/or modifications of histone methylation and/or acetylation (Earley *et al.*, 2010; Mozgova *et al.*, 2010; Pontvianne *et al.*, 2010, 2012, 2013; Durut *et al.*, 2014). None of these features were affected in *nas124* (Fig. 6). In contrast, CHG and CHH methylation on the rDNA promoter were increased in *nas124*. This is unexpected as increased DNA methylation is generally correlated with gene repression (Probst *et al.*, 2004; Earley *et al.*, 2006; Espada *et al.*, 2007). In this context, the conserved iron-sulfur cluster assembly protein MET18 could play a key role. Indeed, in Arabidopsis, *MET18* gene disruption causes DNA hypermethylation in particular in the CHH context (Wang *et al.*, 2016). Interestingly, similarly to *nas124* the rRNA *VAR1* accumulated in *met18* plants (Supplementary Fig. S13). Therefore, in *nas124*, lack of nucleolar iron might inhibit MET18 causing CHH hypermethylation of rDNA.

In animal cells there is a subset of rRNA genes that are transcriptionally inactive but are poised for transcription activation. Such poised rDNA promoters are unmethylated and are marked by both euchromatic and heterochromatic histone modifications (Xie *et al.*, 2012; Grummt and Langst, 2013). Therefore, we can speculate that hypermethylation in *nas124* takes place on ‘poised rDNA promoters’ rather than on transcriptionally active rDNA. Furthermore, higher numbers of FC in *nas124* is a likely consequence of nucleolar association of NOR2 and an increased number of transcribed rRNA genes. However, we predict a low RNA pol I transcription activity in these rRNA genes since accumulation of rRNA precursors and/or mature 18S, 5.8S, and 25S rRNA in *nas124* is similar to WT and *nas234* plants (Supplementary Fig. S14). Controlling rRNA synthesis by modulating RNA pol I loading on rDNA and activity has been reported in yeast and animal cells (French *et al.*, 2003; Goodfellow and Zomerdijk, 2013; Darriere *et al.*, 2019).

NAS enzymes use SAM to produce NA (Fig. 1) and it is possible that reduced rates of NA synthesis in *nas124* and *nas234* might fine-tune SAM availability and subsequently affect methylation of RNA, DNA, and/or proteins. Remarkably, in contrast to the lack of nucleolar iron and NOR2 gene expression detected in *nas124*, RNA methylation modifications are impacted in both *nas124* and *nas234* (Fig. 7). However, the specific and contrasting changes of RNA modifications levels in *nas124* and *nas234* are intriguing. For instance, whereas increased level of RNA modifications (including m^1^A, m^5^C, m^7^G) could be correlated with a differential rRNA gene expression in *nas124*, this is not the case for a decreased level of these same RNA modifications in *nas234.* The identification of differentially methylated RNAs in *nas124* and *nas124* requires further investigation. However, detected modifications m^1^A, m^5^C, m^7^G and ac^4^C are likely associated with rRNA because they are the most abundant cellular RNAs and the first to be detected by LC-MSMS. These modifications occur co-transcriptionally in the nucleolus (Sharma and Lafontaine, 2015; Sloan *et al.*, 2017), and it is tempting to propose that lack of nucleolar iron might affect the activity of iron-sulfur (Fe-S) protein factors involved in depositing RNA modifications. Notably, several rRNA modifications are introduced by RNA modifying enzymes containing (Fe-S) clusters (Kimura and Suzuki, 2015). However, to our knowledge, rRNA modifying enzymes containing (Fe-S) clusters have not yet been reported in plants.

Redox-active iron [Fe^2+^, iron (II)] has the potential to produce a highly reactive^.^OH radicals by the Fenton reaction when it meets hydrogen peroxide (Smethurst and Shcherbik, 2021). However, nuclear/nucleolar redox state seems unaffected in *nas124*, suggesting that nucleolar iron accumulating in WT plants is likely unavailable for Fenton reactions. We did not observe more pronounced ROS accumulation, indicating that *nas124* does not experience oxidative stress due to perturbed cellular/or subcellular iron distribution (Fig. 3; Supplementary Fig. S4). Nevertheless, it is of particular interest to highlight that the ribosome, which generally depends on Mg^2+^, might also contain several Fe^2+^ at specific sites (Smethurst *et al.*, 2020). Therefore, further characterization of *nas124* plants should help us to understand the role of iron in ribosome assembly and/or activity specifically under oxidative conditions.

## Supplementary data

The following supplementary data are available at *JXB* online.

Fig. S1. Identification of triple *nas* mutant plants.

Fig. S2. Nucleolar iron and ferritin in *nas124* and *nuc1* mutant plants.

Fig. S3. Nucleolar iron in roots from *nas124* and *nas234* plants.

Fig. S4. Redox activity in *nas124*.

Fig. S5. Transcriptomic analysis of *nas124*.

Fig. S6. Northern blots to detect long and small non-coding RNA in *nas124* and *nas234*.

Fig. S7. DNA methylation in *nas124*.

Fig. S8. H3 histone marks in *nas124*.

Fig. S9. NOR/ rDNA functional organization in *nas234*.

Fig. S10. RNA methylation changes in *nas124* and *nas234* plants.

Fig. S11. Iron and MS/sucrose impact on *nas124* and *nas234* plants.

Fig. S12. rRNA expression in *nas124* during seedling establishment.

Fig. S13. Expression of rRNA variants in *met18.*

Fig. S14. Transcription and processing of pre-rRNAs in *nas124* and *nas234.*

Table S1. List of primers used in this work.

Table S2. RNAseq analysis of *nas124.*

Table S3. GO analysis of protein genes de-regulated in *nas124.*

Table S4. LC-MRM method data for WT, *nas124*, and *nas234* plants.

Table S5. RiboMethSeq data for WT, *nas124*, and *nas234* plants.

erad180_suppl_Supplementary_Figures_S1-S14

erad180_suppl_Supplementary_Table_S1

erad180_suppl_Supplementary_Table_S2

erad180_suppl_Supplementary_Table_S3

erad180_suppl_Supplementary_Table_S4

erad180_suppl_Supplementary_Table_S5

## Data Availability

All data supporting the findings of this study are available within the paper and within its supplementary materials published online.

## Abbreviations

NA: nicotianamine
NAS: NICOTIANAMINE SYNTHASE
NORs: nucleolar organizer regions
SAM: S-adenosyl-methionine
DAB: 3,3'-diaminobenzidine
ROS: reactive oxygen species
GRX1: glutaredoxin 1
Fib2: fibrillarin 2
NBT: nitro blue tetrazolium chloride
OPT3: OLIGOPEPTIDE TRANSPORTER
IRT3: IRON REGULATED TRANSPORTER 3
TEM: transmission electron microscopy
FC: fibrillar centres
DFC: dense fibrillar component
GC: granular component
ribosomal RNA and DNA: rRNA and rDNA
FISH: fluorescence *in situ* hybridization
ChIP: chromatin immunoprecipitation
LC-MRM: liquid chromatography-multiple reaction monitoring
RiboMethSeq: RNA 2ʹ-*O*-methylation sequencing
