# Supplementary material for: NICOTIANAMINE SYNTHASE activity affects nucleolar iron accumulation and impacts rDNA silencing and RNA methylation in Arabidopsis
Source: J Exp Bot. 2023 May 14;74(15):4384–400. doi: 10.1093/jxb/erad180 (PMC10433931; doi:10.1093/jxb/erad180)
Supplement: erad180_suppl_Supplementary_Figures_S1-S14 [file erad180_suppl_supplementary_figures_s1-s14.pdf]

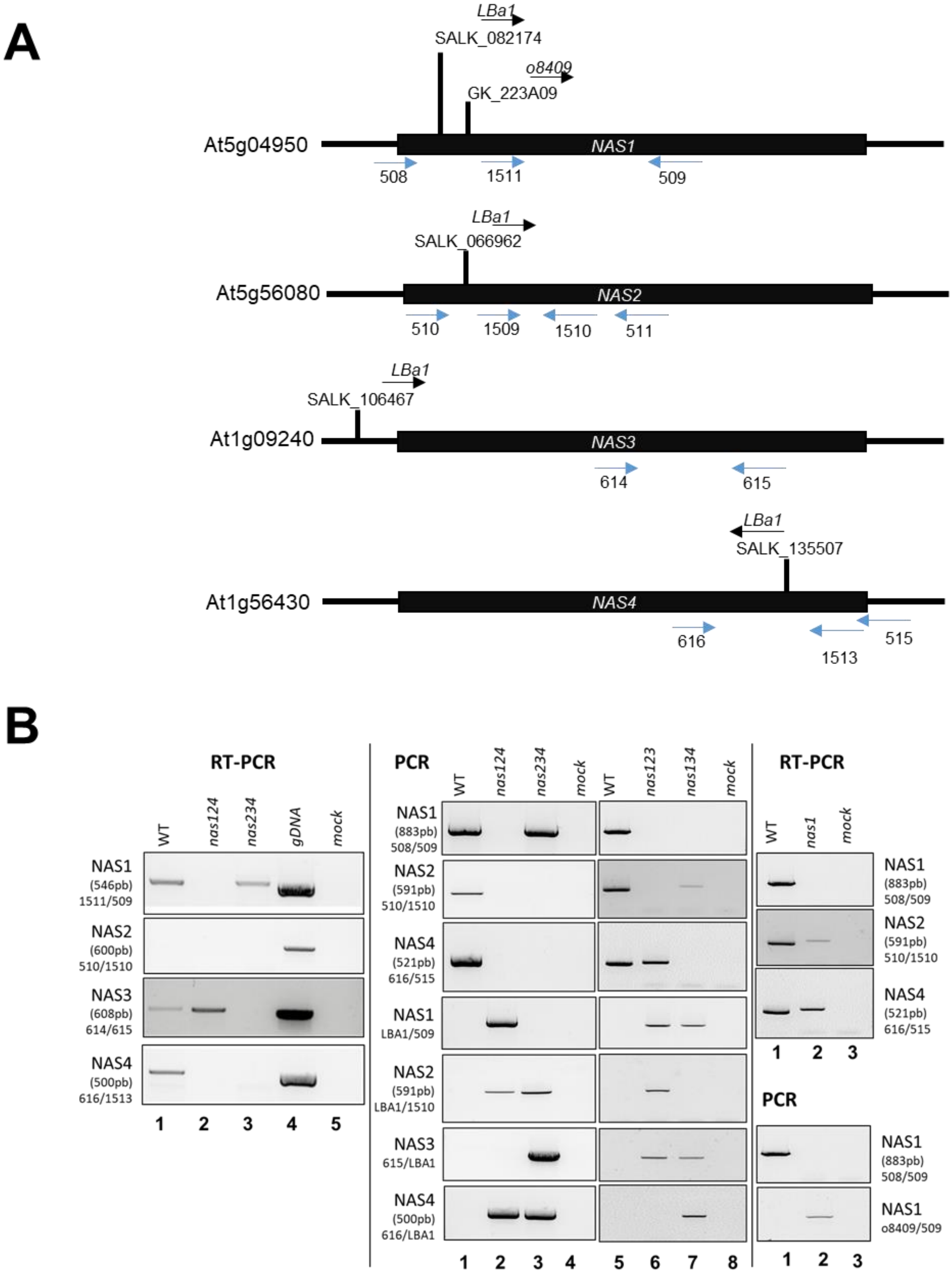

C

	nas123	*nas234*	*nas124*	*nas134*	*nas1*
roots					
leaves					
NAS activity	NAS4	NAS1	NAS3	NAS2	NAS2,3 and 4

Figure S1: Identification of triple *nas* mutant plants A) Genomic DNA structure of the *Arabidopsis thaliana NAS1* (At5g04950*)*, *NAS2* (At5g56080*)*, *NAS3* (At1g09240) and *NAS4* (At1g09240) intro-less genes. The black boxes represent the coding sequences (CDS) and the lines represent the non-coding regions (5' and 3'UTRs). The position of the T-DNA insertions in the respective genes is represented by a vertical bar. The positions of gene specific and T-DNA primers are shown **B)** Left; semi-quantitative RT-PCR using total RNA extracted from 15-days-old WT, *nas124* and *nas234* plants. Middle, PCR amplification using gene specific primers pairs located upstream and downstream of the T-DNA insertion or using primers LBa1 and gene specific located downstream of the T-DNA insertions in *nas124***;** *nas234*; *nas123* and *nas134* triple mutants. Right; Analysis of simple mutant *nas1*. Top, RT-PCR using total RNA extracted from 15-days-old WT and *nas1* plants. Bottom, PCR amplification using gene specific primers pairs located upstream and downstream of the T-DNA insertion or using primer located in the T-DNA insertion and downstream of the T-DNA insertions in *nas1* plants. Genomic amplification of *NAS1, 2,* 3 and *4* were performed to control PCR amplifications with selected primer pairs. Mock lanes are RT and RT-PCR reactions with water instead of genomic DNA or cDNA. Primers indicated in each reaction and expected amplification products are indicated. **C)** NAS gene expression in roots and/or leaves in triple *nas* mutant plants. According to (Bauer *et al.*, 2004). Black, grey and light grey rectangles indicate relative high, medium or low NAS activity in roots or leaves from *nas* mutant plants.

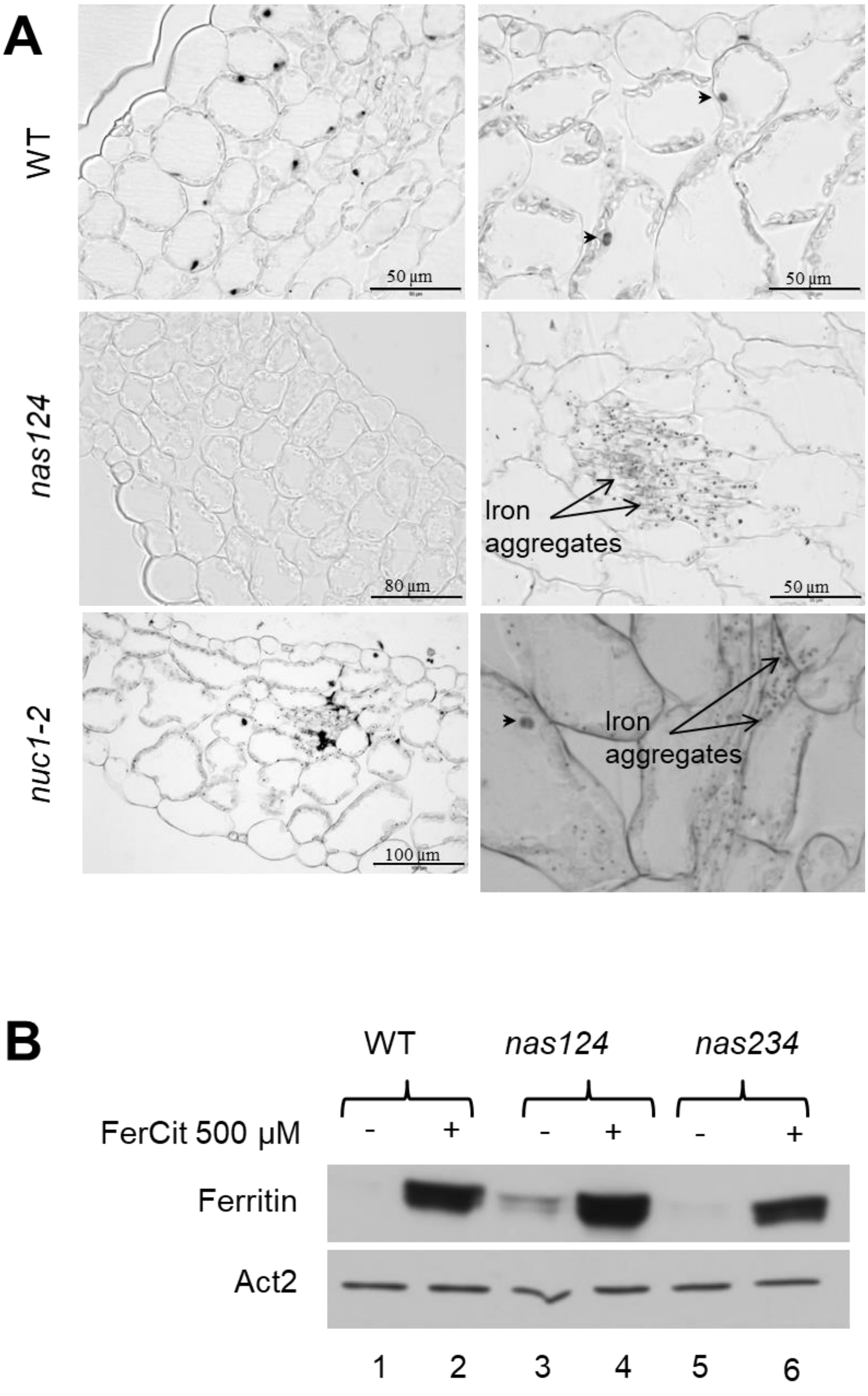

Figure S2: Nucleolar iron and Ferritin in *nas124 and nuc1* mutant plants. A) Epidermal cells from 15 days-old WT, *nas124* and *nuc1-2* plants grown *in vitro* and stained with Perls/DAB. Arrow heads show nucleolar iron and arrows indicate either ferritin spots or iron aggregates. Bar = 50 µm **B)** Western blot analysis of WT; *nas124* and *nas234* plants using antibodies against Arabidopsis ferritin and actin-2 proteins.

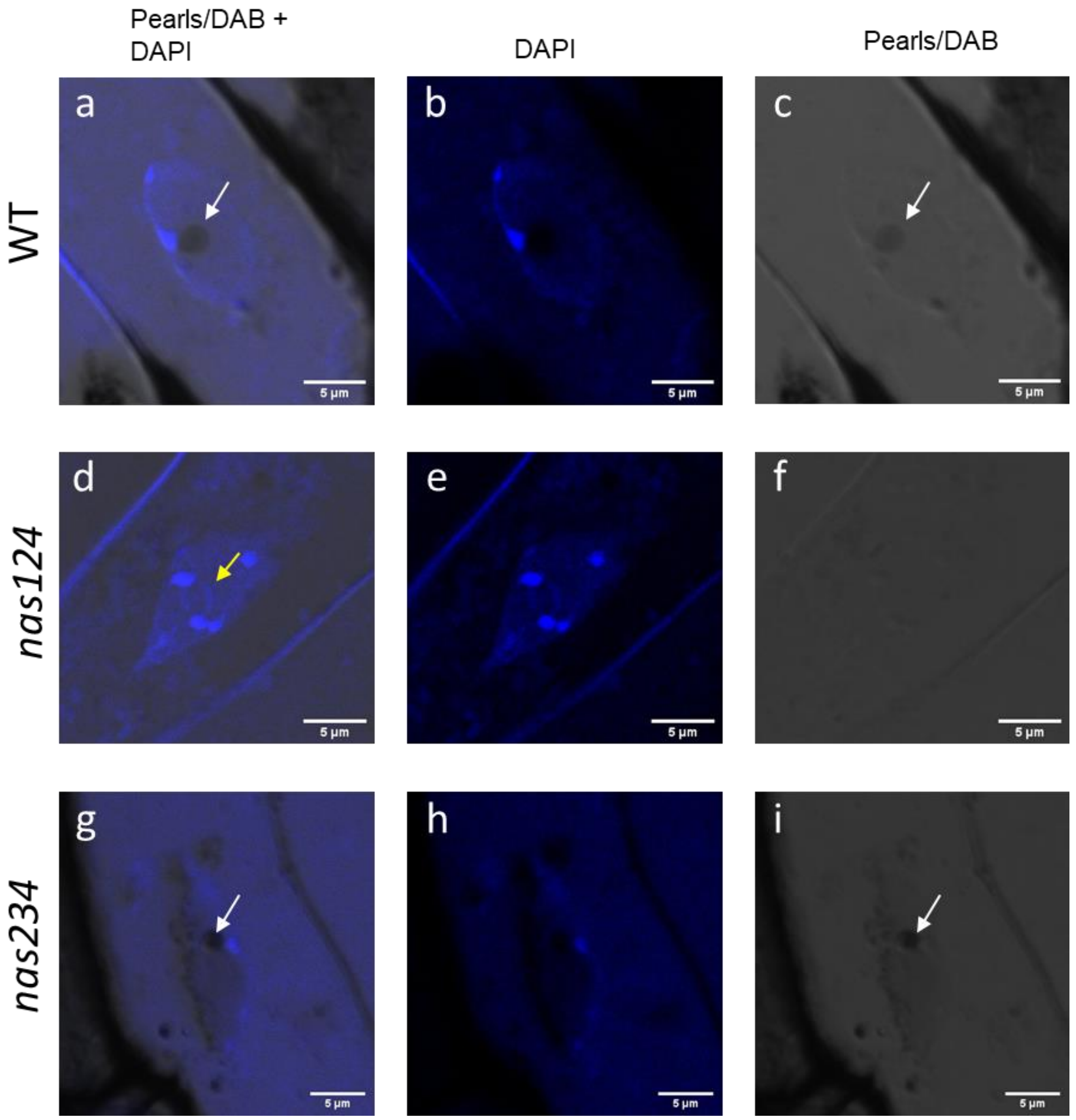

Figure S3: **Nucleolar iron in roots from *nas124* and *nas234*.** Roots sections from 15 days-old WT (a-c), *nas124* (d-f) and *nas234* (g-i) plants grown *in vitro* were stained with Perls/DAB and/or DAPI. White arrows indicate nucleolar iron in WT and *nas234* and yellow arrow show nucleolus without iron in *nas234*. Scale bars = 5 µm.

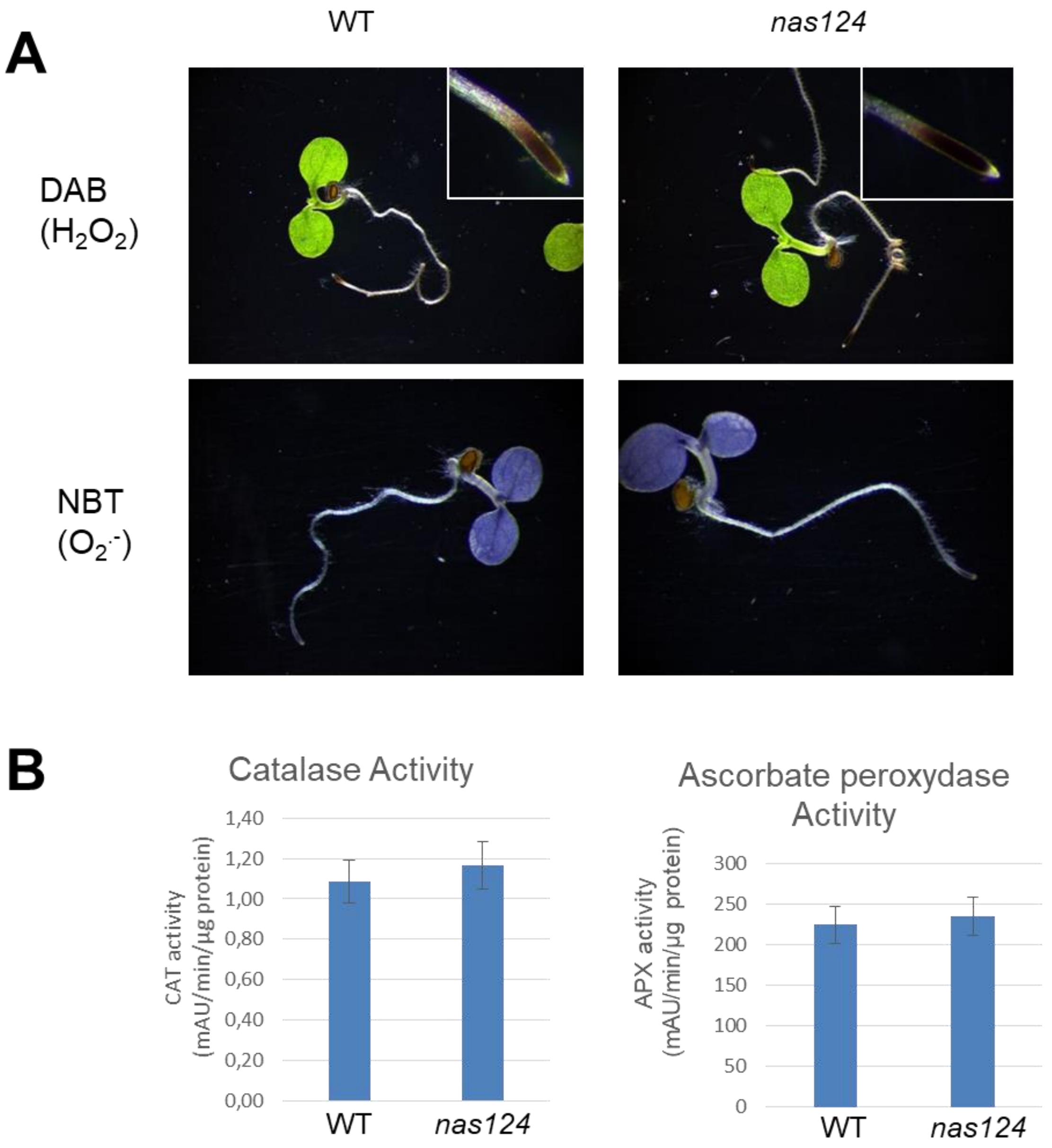

Fenton reaction: $Fe^{2+} + H_2O_2 \rightarrow Fe^{3+} + \cdot OH + OH-$

Figure S4: Redox activity in *nas124.* A) ROS detection in WT and *nas124* plants. Seven-day old plants were subjected to DAB (3,3'-Diaminobenzidine) and NBT (Nitro Blue Tetrazolium) staining, to detect H_2O_2 and $O_2^{\cdot -}$, respectively. Insets focus on the primary root meristem. Representative pictures are shown. N=15 to 20. **B)** Catalase and Ascorbate peroxidase activities in WT and *nas124* plants.

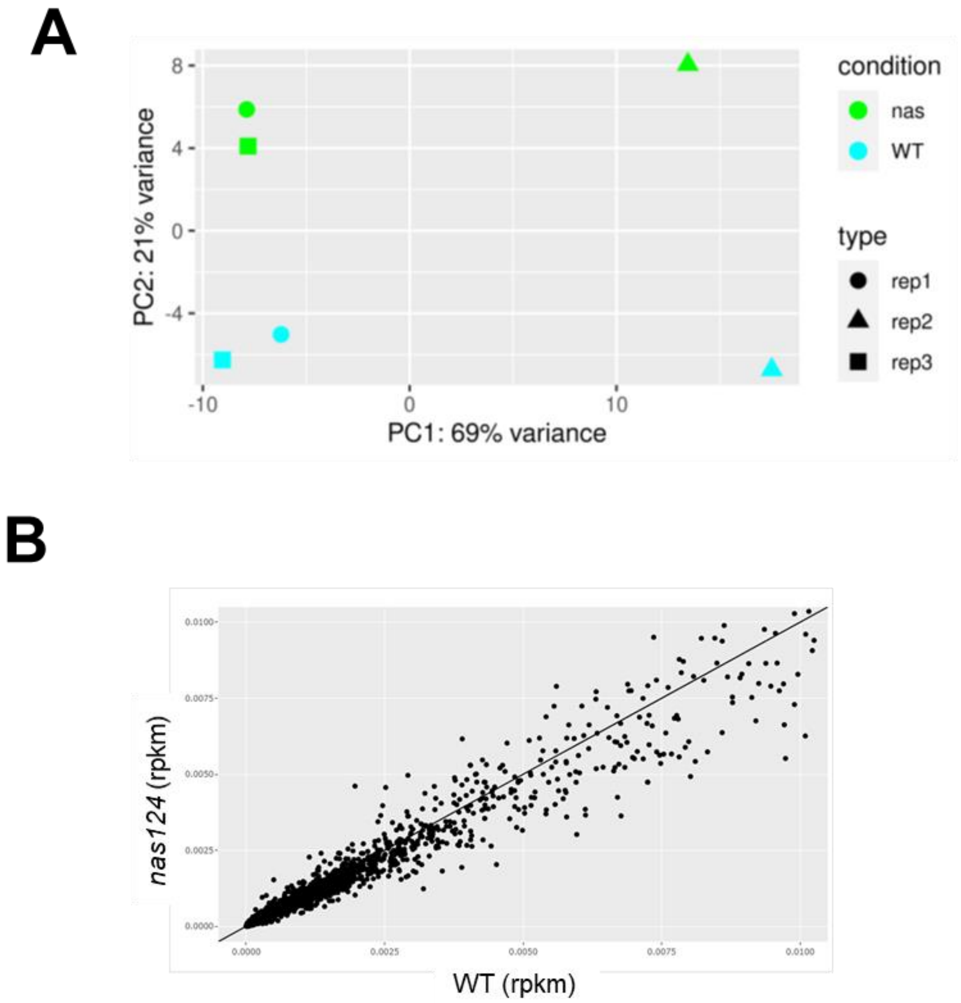

Figure S5: Transcriptomic analysis of *nas124*. A) Principal Component analysis to select the less variable or most reliable WT and *nas124* RNAseq replicates. **B)** Graph of normalized reads (rpkm) in WT and *nas124* plants for long non-coding RNAs. Graphs were generated using RStudio, Ri 386 version 4.1.1.

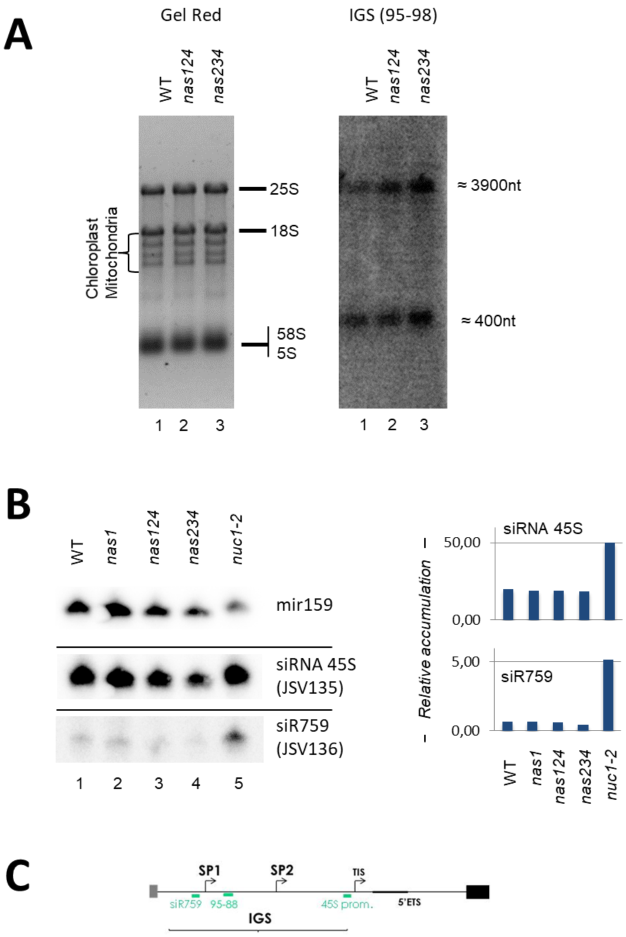

Figure S6: Northern blots to detect long- and small- non-coding RNA in *nas124* and *nas234*. A). Northern blots using total RNA from WT (lane 1), *nas124* (lane 2) and *nas234* (lane 3) plants and probe sequence 95-88. Gel Red is used as RNA loading and size migration controls. The 95-88 probe hybridizes with two IGS RNAs, one around 3900 nucleotides and the second one around 400 nucleotides. This membrane (1) was later hybridized with specific probes p25S and p5 (show in Supplementary Fig. S14B). **B)** Northern blots using total RNA from WT (lane 1), *nas1* (lane 2) and *nas124* (lane 3) *nas234* (lane 4) and *nuc1-2* (lane 5) plants to detect accumulation of promoter siRNAs 45S and R759. RNA from *nuc1-2* was used to control accumulation of siRNA 45S in these plants and hybridization with specific probe for mir159 to estimate relative accumulation of siRNA (Pontvianne *et al.*, 2010). Graph show relative accumulation of siRNA45S, siR759 and tasi255. **C)** Scheme of the 45S InterGenic Sequence (IGS) to show position of the probe sequence 95-88 and oligonucleotides siR45S and oR759 used to detect long ncRNA and small interference RNA (siRNA) transcribed from IGS sequences.

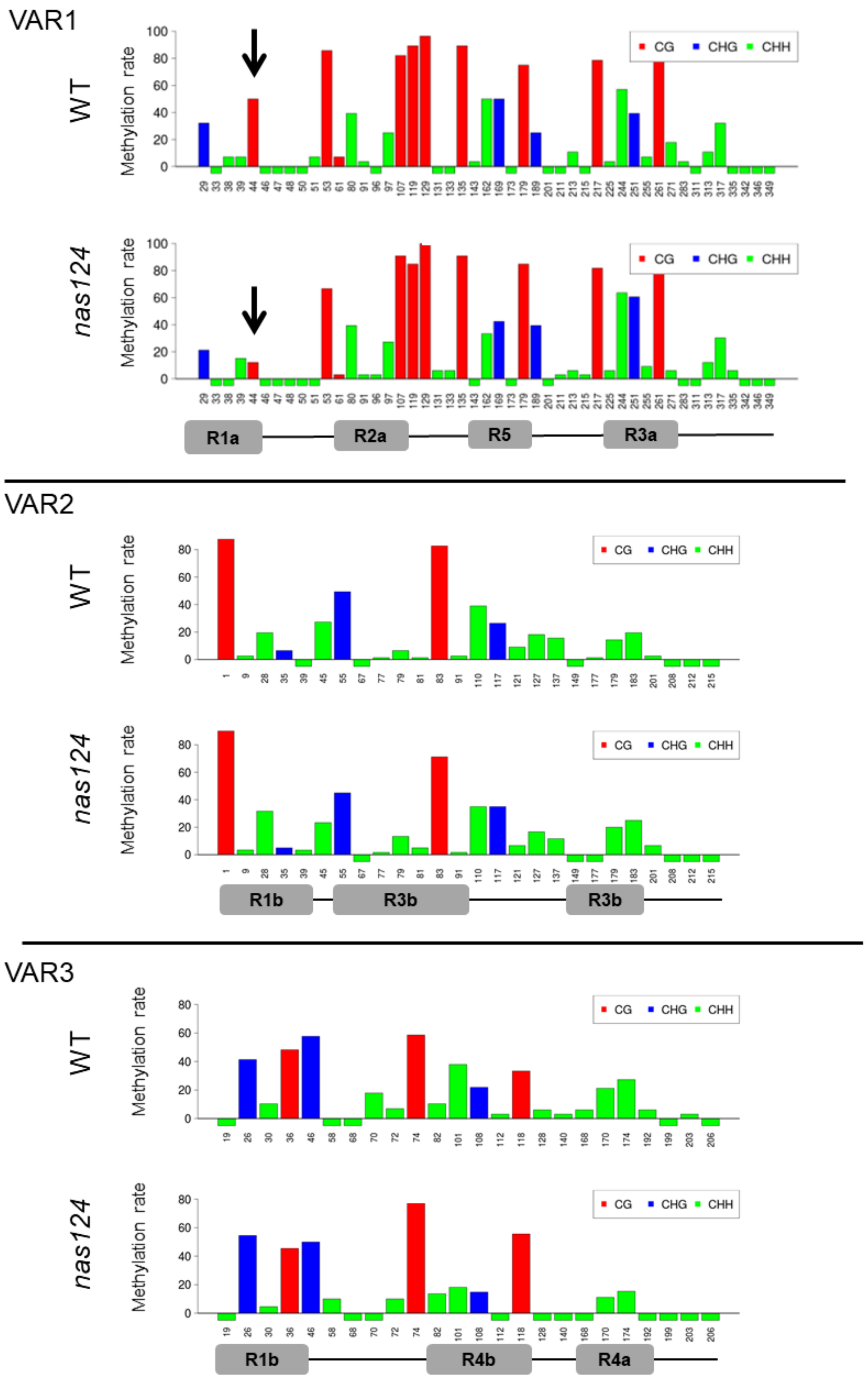

Figure S7: DNA methylation in *nas124*. Bisulfite analysis of 3'ETS VAR1, 2 and 3 from 21 days-old WT and *nas124* plants. The graph bars show CG (red), CHG (blue) and CHH (green) methylation level at different position in the rDNA. The arrow over the graph rDNA VAR1 indicates site with significant (*p* values ≤ 0.05) difference of methylation rate between WT and *nas124* plants.

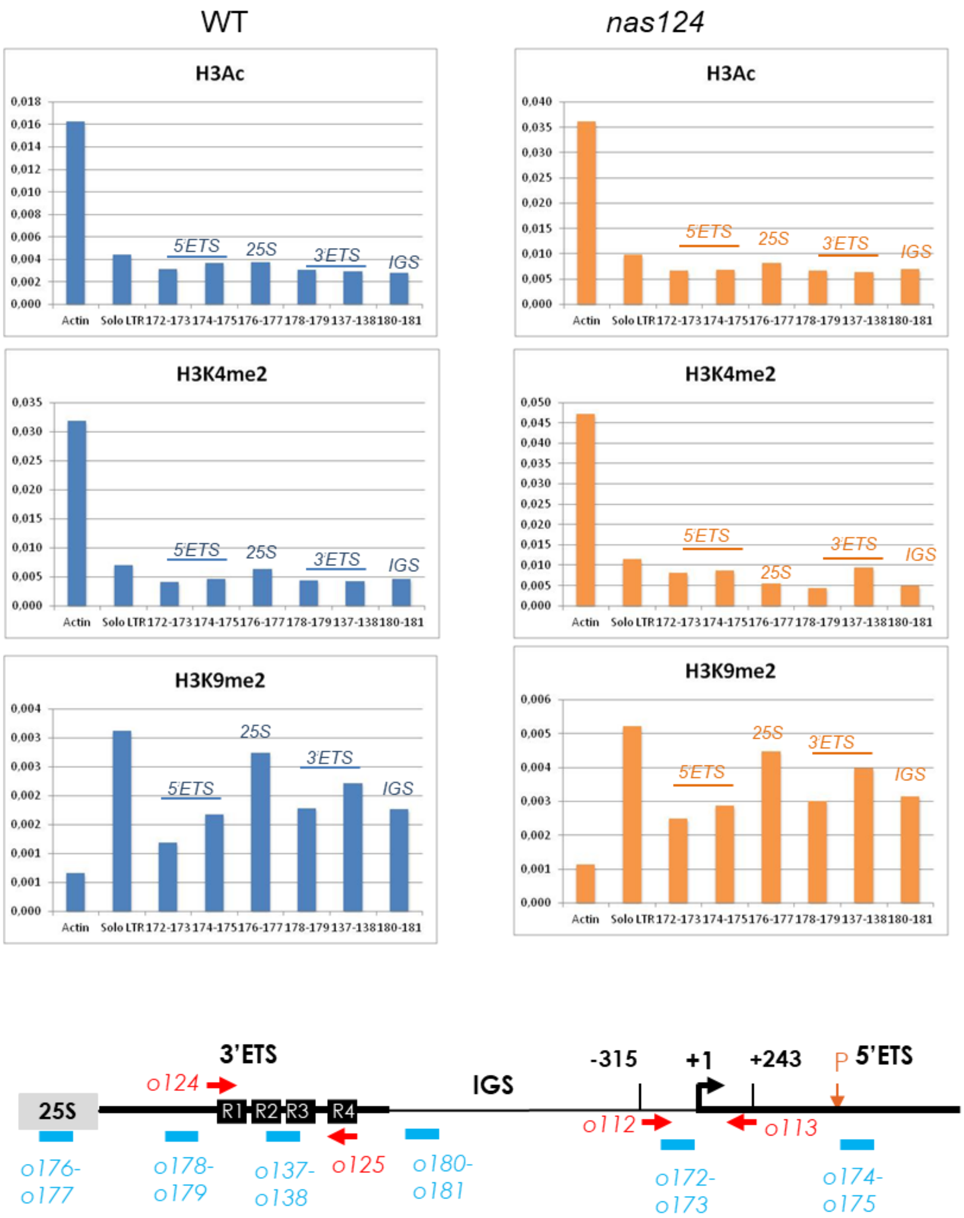

Figure S8: H3 histone marks in *nas124*. ChIP (Chromatin Immuno-Precipitation) was performed using WT (blue) and *nas124* (orange) chromatin and antibodies against permissive (H3K4me2 and H3Ac) and non-permissive (H3K9m2) transcription marks. The histograms show relative enrichment of each mark at specific positions. Amplification of actin and solo LTR serves respectively as permissive and non-permissive chromatin control. Scheme of 45S rDNA shows position of primers used in bisulfite and ChiP analysis. Red arrows, primers *o112/o113* and *o124/o125* for the bisulfite analyses of promoter/5'ETS (from -315 to +243) and 3'ETS rDNA sequences respectively (Supplementary Fig. 6C, S5 and S7). Blue bars, 25S (*o176/o177*), 3'ETS (*o137/o138* and *o178/o179*) IGS (*o180/o181*), promotor (*o172/o173*) and 5'ETS (*o174/o175*) rDNA sequences amplified in ChIP analysis and corresponding oligo primers are indicated. TIS at +1 and P cleavage site in the 5'ETS are shown.

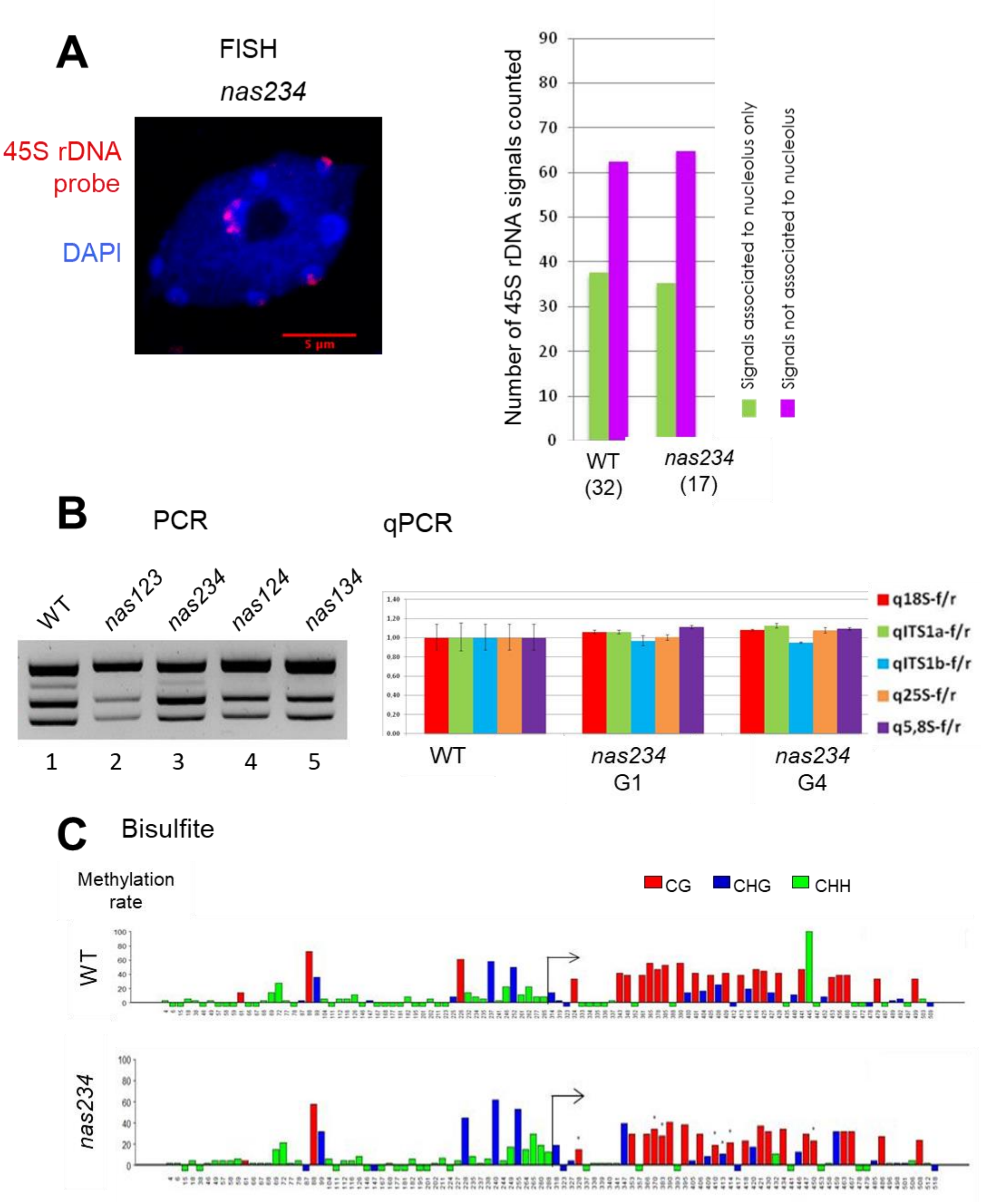

Figure S9: NOR / rDNA functional organization in *nas234* A) FISH analysis in leaf cells from 21 days-old *nas234* plants and histogram show number of 45S signals associated, or not, to the nucleolus in 32 WT and 17 *nas234* cells. The green bar shows number of nucleolus associated signals and the purple bar shows non associated signals **B)** PCR and qPCR to determine ratio of rDNA variants and relative number of 45S rDNA copies in *nas123*, *nas234*; *nas124* and *nas134* and in *nas234* G1 and G4 siblings. PCR lanes 1 (WT) and 4 (*nas124*) are shown separately in Figure 6B bottom panel. **C)** Bisulfite analysis of rDNA promoter and 5'ETS region from *nas234*. The graph bars show CG (red), CHG (blue) and CHH (green) methylation positions. Primers positions for PCR, RT-PCR and bisulfite experiments are depicted in main Figures 6 and Supplementary Fig. S7.

A

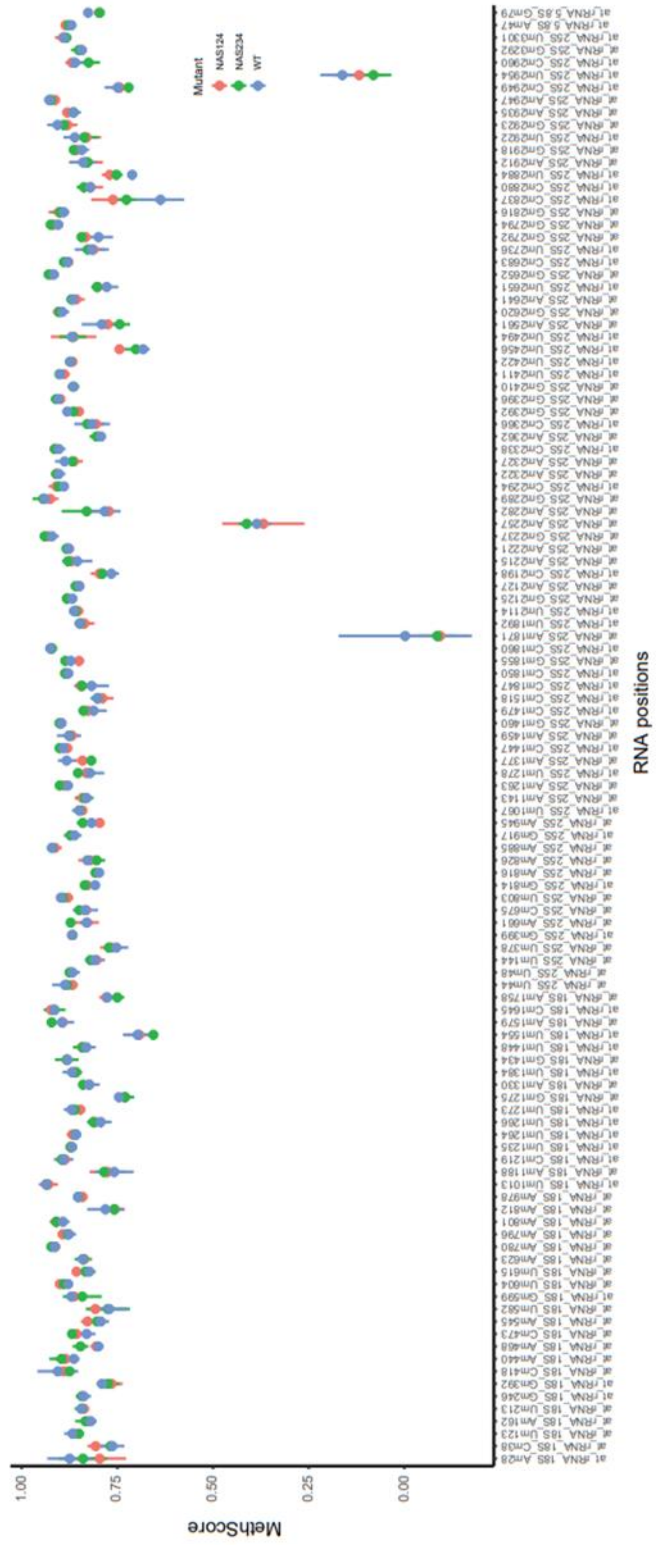
MethScore
RNA positions
Mutant
NAS124
NAS234
WT
1.00
0.75
0.50
0.25
0.00

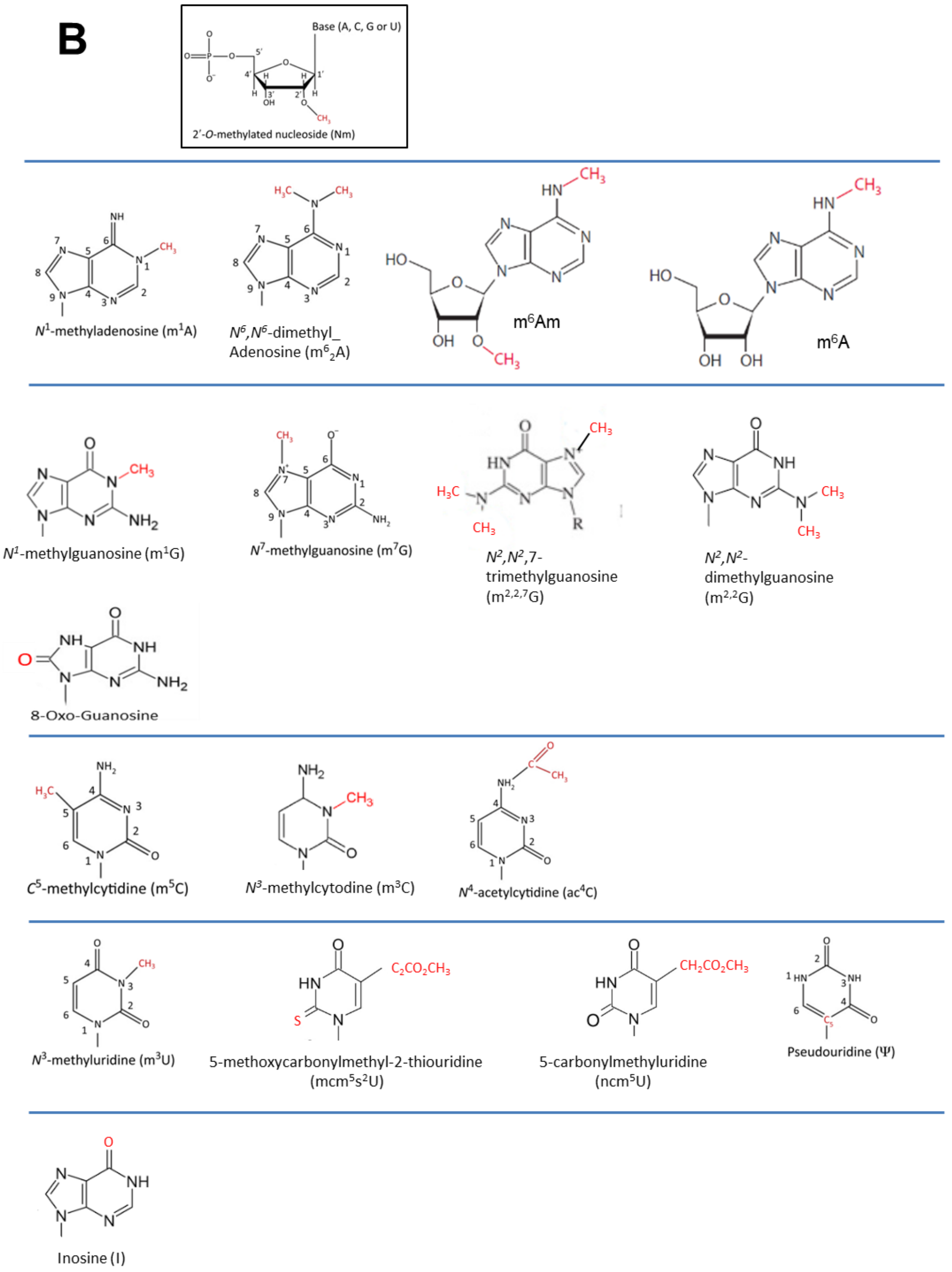

Figure S10. RNA methylation changes in *nas124* and *nas234* plants. A) Total RNAs were extracted from 15 days-old WT, *nas124* and *nas234* plants grown *in vitro* and analyzed by RiboMethSeq. Dotblot representation of methylation score (from three replicas) for each 2'-*O*-Meth sites detected in WT (blue), *nas124* (red) and *nas234* (green) plants. **B)** RNA modifications analyzed LC-MSMS and RiboMethSeq.

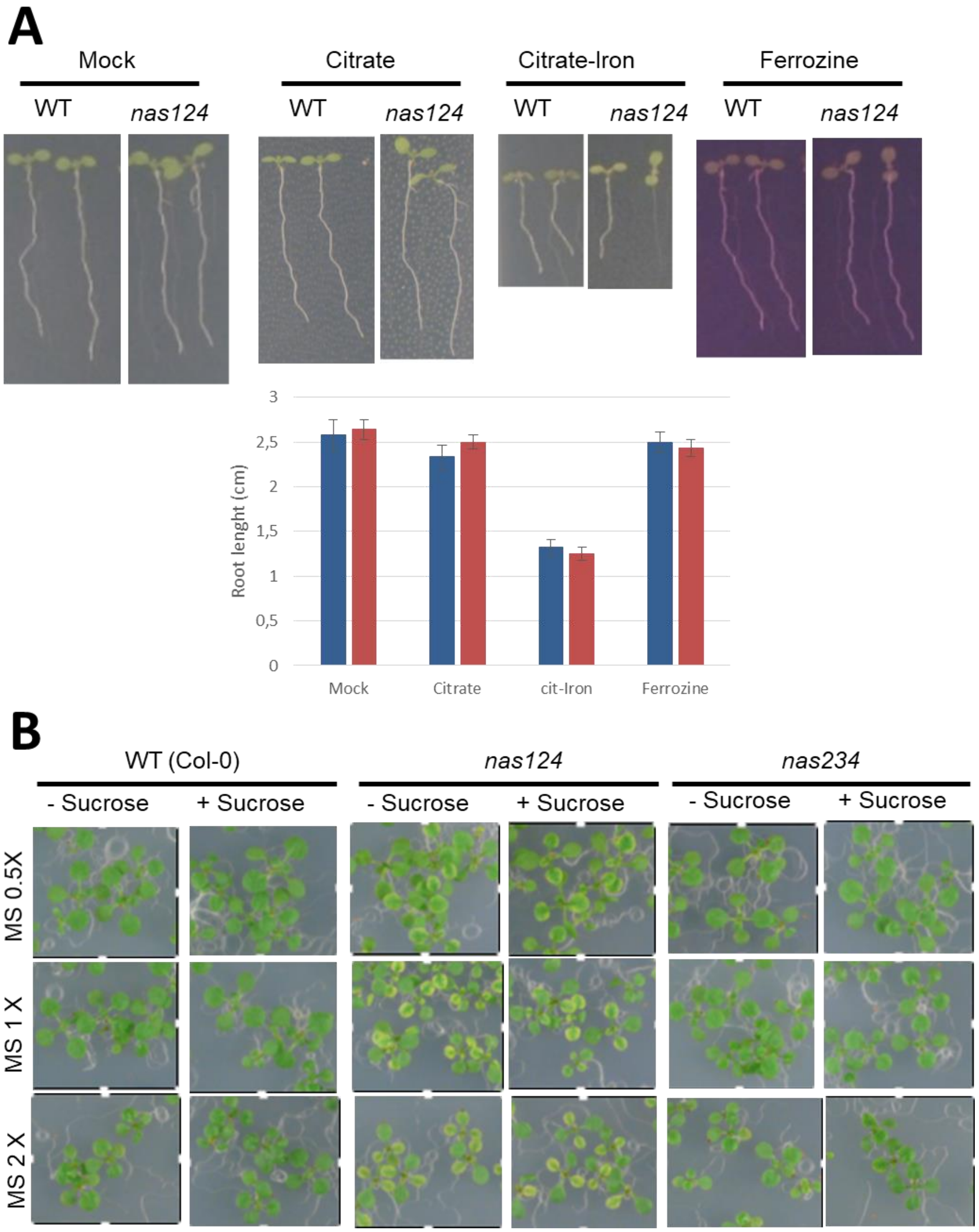

Figure S11: Iron and MS/sucrose impact on *nas124* and *nas234* plants. **A)** WT and *nas124* plants were grown for 15 days on MS1X medium supplemented or not with 2 mM citric acid, 2mM citric acid and 500µM $FeCl_3$ or 100µM Ferrozine. Graphs shows *nas124* roots length at each condition. **B)** WT, *nas124* and *nas234* plants were grown for 15 days on 0.5X; 1X or 2X medium containing or not 1% sucrose.

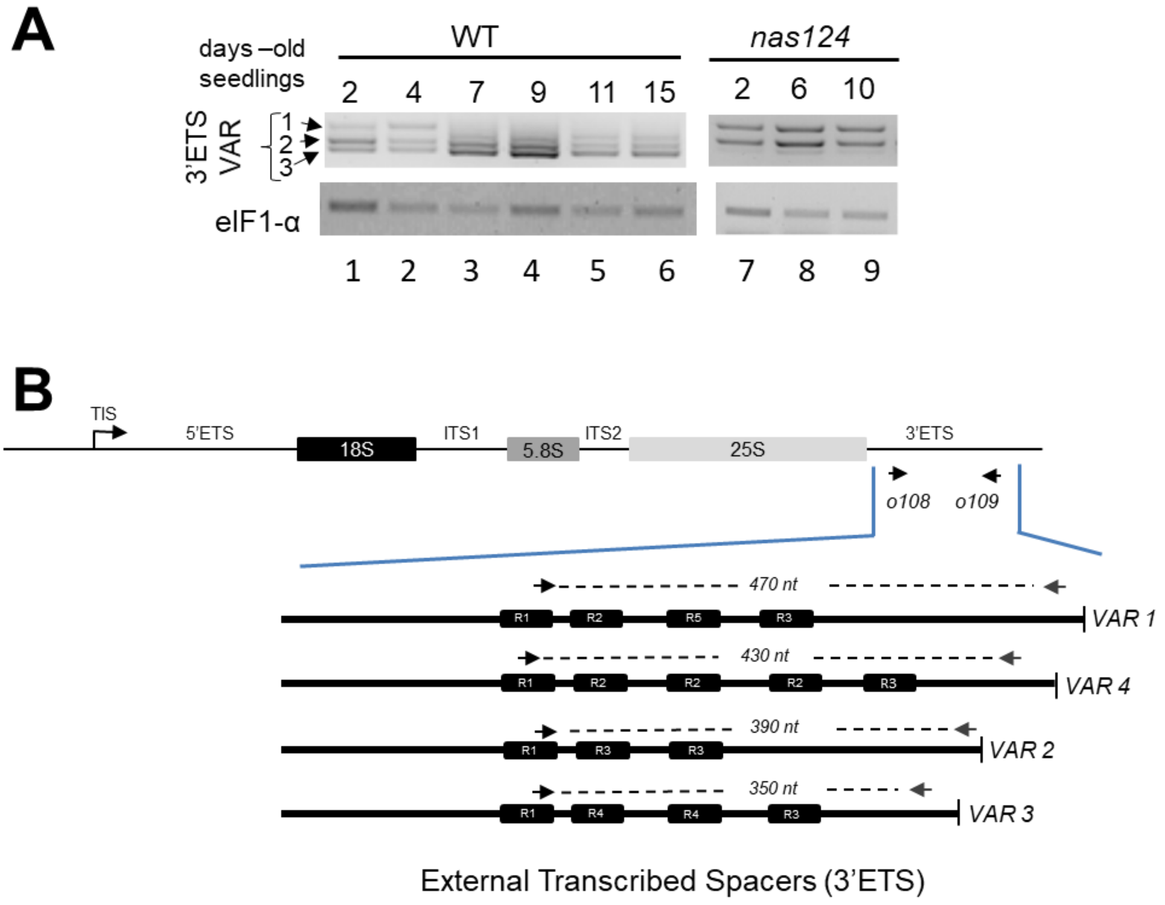

Figure S12: rRNA expression in *nas124* during seedling establishment. A) RT-PCR to detect rRNA variants in WT (2, 4, 7, 9, 11 and 15 days-old) and *nas124* (2, 6, 10 days-old) germinating seedlings. Amplification of EF1-alpha RNA transcripts was performed to verify similar amount of RNA in each sample. **B)** The scheme of a 45S rDNA unit shows position of primers used to detect 3'ETS (o108/o109). The 3'ETS rDNA variants *VAR1*, *2*, *3* and *4* shows repeated boxes (R1-R4), position of primers *o108*/*o109* and expected PCR product sizes for each rRNA variant.

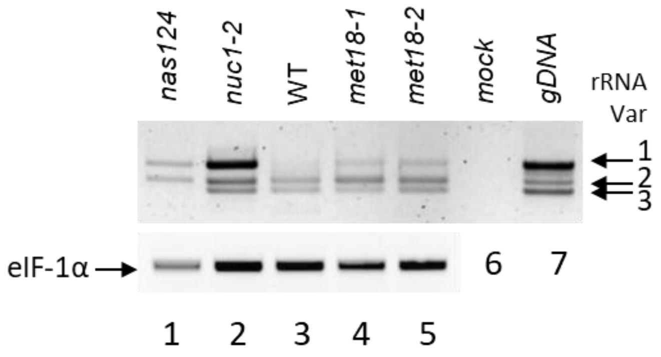

Figure S13: Expression of rRNA variants in *met18*. RT-PCR to detect 45S pre-rRNA variants in leaves from 15 days-old WT, *nas124*, *nuc1-2*, *met18-1* and *met18-2*. Amplification EF1-alpha RNA transcripts was performed to verify similar amount of RNA in each sample. Mutant *met18-1* and *met18-2* plants are SALK_121963C and SALK_147068C respectively.

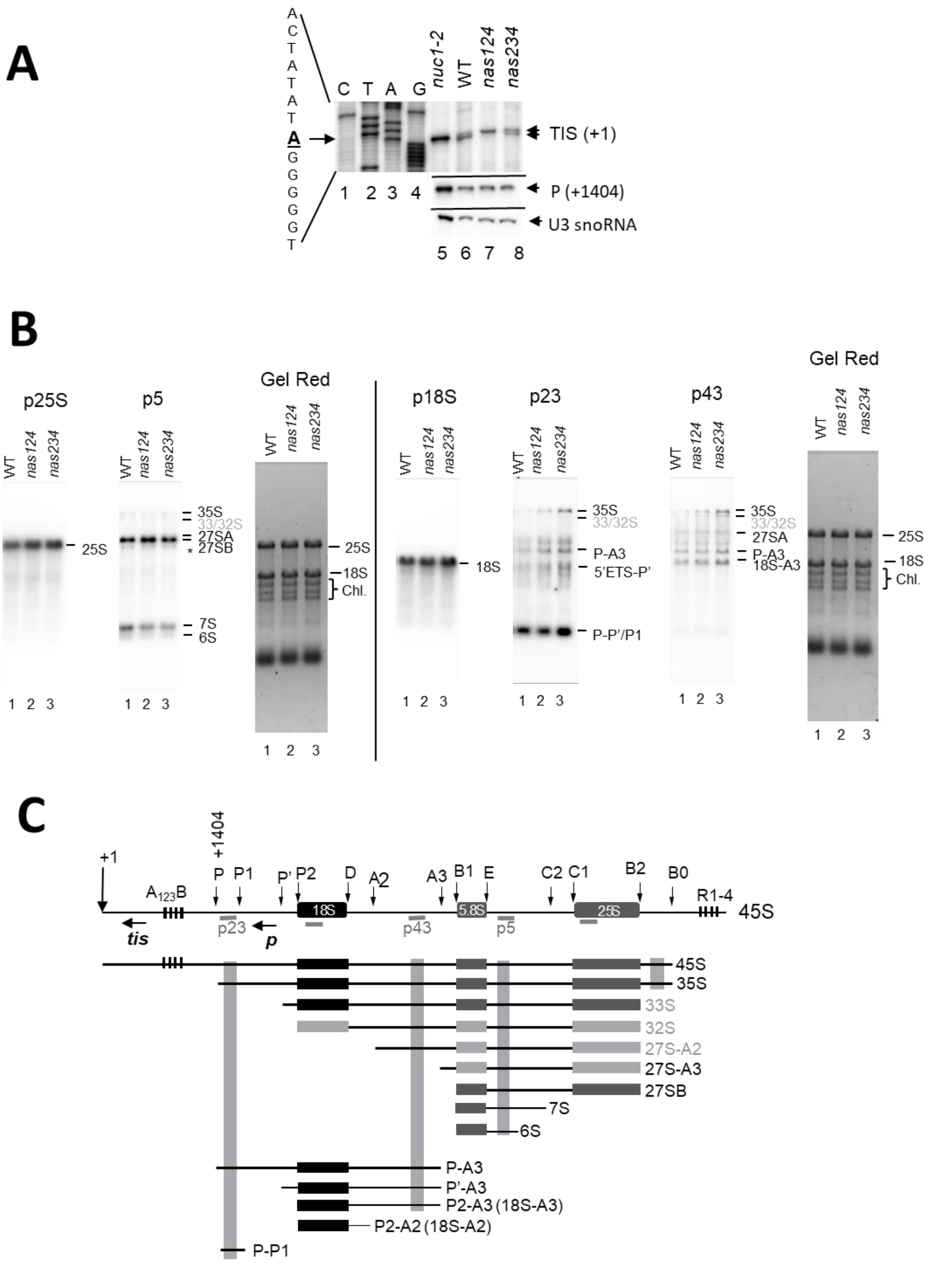

Figure S14: **Transcription and processing of pre-rRNAs in *nas124* and *nas234.* A)** Primer extension using primers *tis* and *p* for mapping TIS and P sites respectively on total RNA from *nuc1-2* (lane 5), WT (lane 6), *nas124* (lane 7) and *nas234* (lane 8) plants. G, A, T and C lanes show DNA sequencing with primer *tis* and used to accurately map transcript initiation site (lanes 1-4). Positions of primers *tis* and *p* respectively at +104 and +1404. TIS at +1 are shown

in C. Primer extension with primer *u3*, was used to detect snoRNA U3 and verify amounts of total RNA in all samples. **B)** Northern blots using total RNA from 21 days-old WT (lane 1), *nas124* (lane 2) and *nas234* (lane 3) plants and oligo primers p5, p23, p43, p18S and p25S. Detected pre-rRNAs are labelled accordingly to previous reports using same probes. Hybridizations with p18S and p25S are used as RNA loading controls and to localize the relative position of rRNAs 18S and 25S. Membrane 1 was hybridized with p5 and then p25S and membrane 2 with p26, p43 and lastly with p18S. The gels stained with red gel and the membranes hybridized with p25S or p18S are used to verify quality and amount of RNAs in each sample. The membrane 1 (p5 and then p25S) was previously hybridized with IGS probe (95-98) shown in Supplementary Fig. S6A. **C)** Scheme representing pre-RNA processing. Specific rRNA transcripts are detected with probes hybridizing 5'ETS (p23), ITS1 (p43), ITS2 (p5), 18S (p18S) and 25S (p25S). rRNA precursors and fragments from major ITS1-first (black labelled) and minor 5'ETS-first (grey labelled) pathways are illustrated.

Bauer, P., Thiel, T., Klatte, M., Bereczky, Z., Brumbarova, T., Hell, R. and Grosse, I. (2004) Analysis of sequence, map position, and gene expression reveals conserved essential genes for iron uptake in Arabidopsis and tomato. *Plant Physiol*, **136**, 4169-4183.

Pontvianne, F., Abou-Ellail, M., Douet, J., Comella, P., Matia, I., Chandrasekhara, C., Debures, A., Blevins, T., Cooke, R., Medina, F.J., Tourmente, S., Pikaard, C.S. and Saez-Vasquez, J. (2010) Nucleolin is required for DNA methylation state and the expression of rRNA gene variants in Arabidopsis thaliana. *PLoS Genet*, **6**, e1001225.
